# The Barker proposal: Combining robustness and efficiency in gradient‐based MCMC

**DOI:** 10.1111/rssb.12482

**Published:** 2022-01-11

**Authors:** Samuel Livingstone, Giacomo Zanella

**Affiliations:** ^1^ Department of Statistical Science University College London UK; ^2^ Department of Decision Sciences BIDSA and IGIER, Bocconi University Milan Italy

**Keywords:** adaptive tuning, Bayesian computation, MCMC, Metropolis–Hastings, spectral gap

## Abstract

There is a tension between robustness and efficiency when designing Markov chain Monte Carlo (MCMC) sampling algorithms. Here we focus on robustness with respect to tuning parameters, showing that more sophisticated algorithms tend to be more sensitive to the choice of step‐size parameter and less robust to heterogeneity of the distribution of interest. We characterise this phenomenon by studying the behaviour of spectral gaps as an increasingly poor step‐size is chosen for the algorithm. Motivated by these considerations, we propose a novel and simple gradient‐based MCMC algorithm, inspired by the classical Barker accept‐reject rule, with improved robustness properties. Extensive theoretical results, dealing with robustness to tuning, geometric ergodicity and scaling with dimension, suggest that the novel scheme combines the robustness of simple schemes with the efficiency of gradient‐based ones. We show numerically that this type of robustness is particularly beneficial in the context of adaptive MCMC, giving examples where our proposed scheme significantly outperforms state‐of‐the‐art alternatives.

## 1 INTRODUCTION

The need to compute high‐dimensional integrals is ubiquitous in modern statistical inference and beyond (e.g. Brooks et al., 2011; Krauth, 2006; Stuart, 2010). Markov chain Monte Carlo (MCMC) is a popular solution, in which the central idea is to construct a Markov chain with a certain limiting distribution and use ergodic averages to approximate expectations of interest. In the celebrated Metropolis–Hastings algorithm, the Markov chain transition is constructed using a combination of a ‘candidate’ kernel, to suggest a possible move at each iteration, together with an accept‐reject mechanism (Hastings, 1970; Metropolis et al., 1953). Many different flavours of Metropolis–Hastings exist, with the most common difference being in the construction of the candidate kernel. In the Random walk Metropolis (RWM), proposed moves are generated using a symmetric distribution centred at the current point. Two more sophisticated methods are the Metropolis‐adjusted Langevin algorithm (Roberts & Tweedie, 1996) and Hamiltonian/hybrid Monte Carlo (Duane et al., 1987; Neal, 2011). Both use gradient information about the distribution of interest (the *target*) to inform proposals. Gradient‐based methods are widely considered to be state‐of‐the‐art in MCMC, and much current work has been dedicated to their study and implementation (e.g. Beskos et al., 2013; Dalalyan, 2017; Durmus & Moulines, 2017).

Several measures of performance have been developed to help choose a suitable candidate kernel for a given task. One of these is high‐dimensional scaling arguments, which compare how the efficiency of the method decays with *d*, the dimension of the state space. For the random walk algorithm this decay is of the order $d^{-1}$ (Roberts et al., 1997), while for the Langevin algorithm the same figure is $d^{-1/3}$ (Roberts & Rosenthal, 1998) and for Hamiltonian Monte Carlo (HMC) it is $d^{-1/4}$ (Beskos et al., 2013). Another measure is to find general conditions under which a kernel will produce a geometrically ergodic Markov chain. For the random walk algorithm, this essentially occurs when the tails of the posterior decay at a faster than exponential rate and are suitably regular (more precise conditions are given in Jarner & Hansen, 2000). The same is broadly true of the Langevin and Hamiltonian schemes (Durmus et al., 2017a; Livingstone et al., 2019; Roberts & Tweedie, 1996), but here there is an additional restriction that the tails should not decay too quickly. This limitation is caused by the way in which gradients are used to construct the candidate kernel, which can result in the algorithm generating unreasonable proposals that are nearly always rejected in certain regions (Livingstone et al., 2019; Roberts & Tweedie, 1996).

There is clearly some tension between the different results presented above. According to the scaling arguments, gradient information is preferable. The ergodicity results, however, imply that gradient‐based schemes are typically less *robust* than others, in the sense that there is a smaller class of limiting distributions for which the output will be a geometrically ergodic Markov chain. It is natural to wonder whether it is possible to incorporate gradient information in such a way that this measure of robustness is not compromised. Simple approaches to modifying the Langevin algorithm for this purpose have been suggested (based on the idea of truncating gradients, for example Atchade, 2006; Roberts & Tweedie, 1996), but these typically compromise the favourable scaling of the original method. In addition to this, it is often remarked that gradient‐based methods can be difficult to tune. Algorithm performance is often highly sensitive to the choice of scale within the proposal (Neal, 2003, Figure 15), and if this is chosen to be too large in certain directions then performance can degrade rapidly. Because of this, practitioners must spend a long time adjusting the tuning parameters to ensure that the algorithm is running well, or develop sophisticated adaptation schemes for this purpose (e.g. Hoffman & Gelman, 2014), which can nonetheless still require a large number of iterations to find good tuning parameters (see Sections 5 and 6). We will refer to this issue as *robustness to tuning*.

In this article, we present a new gradient‐based MCMC scheme, *the Barker proposal*, which combines favourable high‐dimensional scaling properties with favourable ergodicity and robustness to tuning properties. To motivate the new scheme, in Section 2, we present a direct argument showing how the spectral gaps for the random walk, Langevin and Hamiltonian algorithms behave as the tuning parameters are chosen to be increasingly unsuitable for the problem at hand. In particular, we show that the spectral gaps for commonly used gradient‐based algorithms decay to zero exponentially fast in the degree of mismatch between the scales of the proposal and target distributions, while for the random walk Metropolis the decay is polynomial. In Section 3, we derive the Barker proposal scheme beginning from a family of *π*‐invariant continuous‐time jump processes, and discuss its connections to the concept of ‘locally balanced’ proposals, introduced in (Zanella, 2020) for discrete state spaces. The name *Barker* comes from the particular choice of ‘balancing function’ used to uncover the scheme, which is inspired by the classical Barker accept‐reject rule (Barker, 1965). In Section 4, we conduct a detailed analysis of the ergodicity, scaling and robustness properties of this new method, establishing that it shares the favourable robustness to tuning of the random walk algorithm, can be geometrically ergodic in the presence of very light tails, and enjoys the $d^{-1/3}$ scaling with dimension of the Langevin scheme. The theory is then supported by an extensive simulation study in Sections 5 and 6, including comparisons with state‐of‐the‐art alternative sampling schemes, which highlights that this kind of robustness is particularly advantageous in the context of adaptive MCMC. The code to reproduce the experiments is available from the online repository at the link https://github.com/gzanella/barker. Proofs and further numerical simulations are provided in the supplement.

### 1.1 Basic set‐up and notation

Throughout we work on the Borel space $\left(R^{d},\;B\right)$, with *d* ≥ 1 indicating the dimension. For λ∈R, we write *λ* ↑ ∞ and *λ* ↓ 0 to emphasize the direction of convergence when this is important. For two functions f,g:R→R, we use the Bachmann–Landau notation f(t)=O(g(t)) if ${\text{lim sup}}_{t\to \infty }\;f\left(t\right)/g\left(t\right)<\infty$ and *f*(*t*) = *Θ*(*g*(*t*)) if both ${\text{lim inf}}_{t\to \infty }\;f\left(t\right)/g\left(t\right)>0$ and f(t)=O(g(t)).

The Markov chains we consider will be of the Metropolis–Hastings type, meaning that the *π*‐invariant kernel *P* is constructed as $P\left(x,\;dy\right):=\alpha \left(x,y\right)Q\left(x,dy\right)+r\left(x\right)\delta_{x}\left(dy\right)$, where $Q:R^{d}\times B\to \left[0,1\right]$ is a candidate kernel,

(1) $$\alpha \left(x,y\right):=\min\left(1,,,\frac{\pi (dy)Q(y,dx)}{\pi (dx)Q(x,dy)}\right)$$

is the *acceptance rate* for a proposal *y* given the current point *x* (provided that the expression is well‐defined, see Tierney, 1998 for details here), and *r*(*x*) := 1−∫*α*(*x*,*y*)*Q*(*x*,*dy*) is the average probability of rejection given that the current point is *x*.

## 2 ROBUSTNESS TO TUNING

In this section, we seek to quantify the robustness of the random walk, Langevin and Hamiltonian schemes with respect to the mismatch between the scales of *π*(·) and *Q* in a given direction. Unlike other analyses in the MCMC literature (e.g. Beskos et al., 2018; Roberts & Rosenthal, 2001), we are interested in studying how MCMC algorithms perform when they are *not* optimally tuned, in order to understand how crucially performance depends on such design choices (e.g. the choice of proposal step‐size or pre‐conditioning matrix). The rationale for performing such an analysis is that achieving optimal or even close to optimal tuning can be extremely challenging in practice, especially when *π*(·) exhibits substantial heterogeneity. This is typically done using past samples in the chain to compute online estimates of the average acceptance rate and the covariance of *π* (or simply its diagonal terms for computational convenience), and then using those estimates to tune the proposal step‐sizes in different directions (Andrieu & Thoms, 2008). If the degree of heterogeneity is large, it can take a long time for certain directions to be well‐explored, and hence for the estimated covariance to be representative and the tuning parameters to converge.

In such settings, algorithms that are more robust to tuning are not only easier to use when such tuning is done manually by the user, but can also greatly facilitate the process of learning the tuning parameters *adaptively* within the algorithm. We show in Sections 5 and 6 that if an algorithm is robust to tuning then this adaptation process can be orders of magnitude faster than in the alternative case, drastically reducing the overall computational cost for challenging targets. The intuition for this is that more robust algorithms will start performing well (i.e. sampling efficiently) earlier in the adaptation process (when tuning parameters are not yet optimally tuned), which in turn will speed up the exploration of the target and the learning of the tuning parameters.

### 2.1 Analytical framework

The most general scenario we consider is a family of target densities $\pi^{\left(\lambda ,k\right)}$ indexed by *λ* > 0 and *k* ∈ {1, …, *d*} defined as

(2) $$\pi^{\left(\lambda ,k\right)}\left(x\right):=\lambda^{-k}\pi \left(x_{1}/\lambda ,\ldots ,x_{k}/\lambda ,x_{k+1},\ldots ,x_{d}\right),\;x=\left(x_{1},\ldots ,x_{d}\right)\in R^{d},$$

where *π* is a density defined on $R^{d}$ for which *π*(*x*) > 0 for all $x\in R^{d}$ and $\log\;\pi \in C^{1}\left(R^{d}\right)$. The set‐up allows modification of the scale of the first *k* components of $\pi^{\left(\lambda ,k\right)}$ through the parameter *λ*. Our main results are presented for the case *k* = 1, and we write $\pi^{\left(\lambda \right)}:=\pi^{\left(\lambda ,1\right)}$ for simplicity, before discussing extensions to the *k* > 1 setting in Section 2.5. We consider targeting $\pi^{\left(\lambda \right)}$ using a Metropolis–Hastings algorithm with fixed tuning parameters, and study performance as *λ* varies. Intuitively, we can think of *λ* as a parameter quantifying the level of heterogeneity in the problem. As a concrete example, consider a RWM algorithm in which given the current state $x^{\left(t\right)}$ the candidate move is $y=x^{\left(t\right)}+\sigma \xi$, with *σ* > 0 a fixed tuning parameter and $\xi ∼N(0,I_{d})$, where $I_{d}$ is the *d* × *d* identity matrix. It is instructive to take *σ* as the optimal choice of global scale for *π*, meaning when *λ* is far from one then *σ* is no longer a suitable choice for the first coordinate of $\pi^{\left(\lambda \right)}$.

In the context of the above, the *λ* ↓ 0 regime is representative of distributions in which one component (in this case the first) has a very small scale compared to all others. Conversely, the *λ* ↑ ∞ regime reflects the case in which one component has a much larger scale than its counterparts. Studying robustness to tuning in the context of heterogeneity is particularly relevant, as highlighted above, as this is exactly the context in which tuning is more challenging. The *λ* ↓ 0 regime is particularly interesting and has been recently considered in Beskos et al. (2018), where the authors study the behaviour of the RWM for ‘ridged’ densities for different values of *k* using a diffusion limit approach. The focus in that work, however, was on the finding optimal tuning parameters for the algorithm as a function of *λ*, whereas the present paper is concerned with the regime in which the tuning parameters are fixed (as discussed above).

The above framework could be equivalently formulated by keeping the target distribution *π* fixed and instead rescaling the first component of the candidate kernel by a factor 1/*λ*. This is indeed the formulation we mostly use in the proofs of our theoretical results. A proof of the mathematical equivalence between the two formulations can be found in the supplement.

### 2.2 Measure of performance

Our measure of performance for the various algorithms will be the spectral gap of the resulting Markov chains. Consider the space of functions

$$L_{0,1}^{2}(\pi )=\{f:R^{d}\to R|E_{\pi }[f]=0,\;{\mathrm{Var}}_{\pi }[f]=1\}.$$

Note that any function *g* with $E_{\pi }g^{2}<\infty$ can be associated with an $f\in L_{0,1}^{2}\left(\pi \right)$ through the map $f=\left(g-E_{\pi }g\right)/\surd {\mathrm{Var}}_{\pi }g$, and that if $X^{\left(t\right)}∼\pi \left(\cdot \right)$ and $X^{\left(t+1\right)}|X^{\left(t\right)}∼P\left(X^{\left(t\right)},\cdot \right)$ then $\text{Corr}\left\{g\left(X^{\left(t\right)}\right),g\left(X^{\left(t+1\right)}\right)\right\}=\text{Corr}\left\{f\left(X^{\left(t\right)}\right),f\left(X^{\left(t+1\right)}\right)\right\}$. The (right) spectral gap of a *π*‐reversible Markov chain with transition kernel *P* is

(3) $$\text{Gap}\left(P\right)=\underset{f\in L_{0,1}^{2}\left(\pi \right)}{\inf}\;\frac{1}{2}\int {\left(f\left(y\right)-f\left(x\right)\right)}^{2}\pi \left(dx\right)P\left(x,dy\right).$$

The expression inside the infimum is called a *Dirichlet form*, and can be thought of as the ‘expected squared jump distance’ for the function *f* provided the chain is stationary. This can in turn be re‐written as $1-\text{Corr}\{f\left(X^{\left(t\right)}\right),f\left(X^{\left(t+1\right)}\right)\}$. Maximising the spectral gap of a reversible Markov chain can therefore be understood as minimising the *worst‐case* first‐order autocorrelation among all possible square‐integrable test functions.

The spectral gap allows to bound the variances of ergodic averages (see Proposition 1 of Rosenthal, 2003). Also, a direct connection between the spectral gap and mixing properties of the chain can be made if the operator *Pf*(*x*) := ∫*f*(*y*)*P*(*x*,*dy*) is positive on $L^{2}\left(\pi \right)$. This will always be the case if the chain is made lazy, which is the approach taken in Woodard et al. (2009), and the same adjustment can be made here if desired.

### 2.3 The small *λ* regime

In this section, we assess the robustness to tuning of the random walk, Langevin and Hamiltonian schemes as *λ* ↓ 0. This corresponds to the case in which the proposal scale is chosen to be too large in the first component of $\pi^{\left(\lambda \right)}$. The results in this section will support the idea that classical gradient‐based schemes pay a very high price for any direction in which this tuning parameter is chosen to be too large, as already noted in the literature (e.g. Neal, 2003, p. 738), while the RWM is less severely affected by such issues.

#### 2.3.1 Random walk Metropolis

In the RWM, given a current point $x\in R^{d}$, a proposal *y* is calculated using the equation

(4) y=x+σξ,

with *σ* > 0 and *ξ* ∼ *μ*(·) for some centred symmetric distribution *μ*. The resulting candidate kernel $Q^{R}$ is given by $Q^{R}\left(x,dy\right)=q^{R}\left(x,y\right)dy$ with $q^{R}\left(x,y\right)=\sigma^{-d}\mu \left(\left(y-x\right)/\sigma \right)$, where *μ*(*ξ*) for $\xi \in R^{d}$ denotes the density of *μ*. Following Section 2.1, we consider Metropolis–Hastings algorithms with proposal $Q^{R}$ and target distribution $\pi^{\left(\lambda \right)}$ defined in Equation (2), and denote the resulting transition kernels as $P_{\lambda }^{R}$.

We impose the following mild regularity conditions on the density *μ*(*ξ*). These are satisfied for most popular choices of *μ*, as shown in the subsequent proposition.

Condition 1 There exists $\lambda_{0}>0$ such that for any $x,y\in R^{d}$ and $\lambda <\lambda_{0}$ we have $\mu \left(\delta_{\lambda }\right)\geq \mu \left(\delta \right)$, where *δ* = *y* − *x* and

(5) $$\delta_{\lambda }:=\left(\lambda \left(y_{1}-x_{1}\right),y_{2}-x_{2},\ldots ,y_{d}-x_{d}\right).$$

In addition, $\sup_{\xi_{1}\in R}\;\mu_{1}\left(\xi_{1}\right)<\infty$, where $\mu_{1}\left(\xi_{1}\right)=\int_{R^{d-1}}\mu \left(\xi_{1},\xi_{2},\ldots ,\xi_{d}\right)d\xi_{2}\ldots d\xi_{d}$ is the marginal distribution of $\xi_{1}$ under *ξ* ∼ *μ*.

Proposition 1 *Denoting the usual* *p*‐*norm as* ${\left\|x\right\|}_{p}={\left(\sum_{i=1}^{d}x_{i}^{p}\right)}^{1/p}$, *Condition* 1 *holds in each of the below cases:*

(i) $q^{R}\left(x,y\right)={\left(2\pi \sigma^{2}\right)}^{‐d/2}{\;\exp(‐\|x‐y\|}_{2}^{2}/\left(2\sigma^{2}\right))$ *(Gaussian)*
(ii) $q^{R}\left(x,y\right)=2^{‐d}{\;\exp(‐\|x‐y\|}_{1})$ *(Laplace)*
(iii) $q^{R}\left(x,y\right)\propto {\left(1+\|y‐x\right\|}_{2}^{2}{/\nu )}^{‐(\nu +d)/2}$ *for* *ν* ∈ {1, 2, …} *(Student's* *t*)

We conclude the section with a characterization of the rate of convergence to zero of the spectral gap for the RWM as *λ* ↓ 0.

Theorem 1 *Assume Condition* 1 *and* $\text{Gap}(P_{1}^{R})>0$. *Then it holds that*

$$\text{Gap}\left(P_{\lambda }^{R}\right)=\Theta \left(\lambda \right),\;\text{as}\;\lambda ↓0.$$

Note that Theorem 1 requires very few assumptions on the target *π* other than $\text{Gap}(P_{1}^{R})>0$. Note also that the lower bound is of the form $\text{Gap}\left(P_{\lambda }^{R}\right)\geq \lambda \text{Gap}\left(P_{1}^{R}\right)$, see proof of Theorem 1 for details. No dependence on the dimension of the problem other than that intrinsic to $\text{Gap}(P_{1}^{R})$ is therefore introduced.

#### 2.3.2 The Langevin algorithm

In the Langevin algorithm (or more specifically the Metropolis‐adjusted Langevin algorithm, MALA), given the current point $x\in R^{d}$, a proposal *y* is generated by setting

(6) $$y=x+\frac{\sigma^{2}}{2}\nabla \;\log\;\pi^{\left(\lambda \right)}\left(x\right)+\sigma \xi ,$$

for some *σ* > 0 and $\xi ∼N(0,I_{d})$. In this case the proposal is no longer symmetric and so the full Hastings ratio (1) must be used. The proposal mechanism is based on the overdamped Langevin stochastic differential equation $dX_{t}=\nabla \;\log\pi^{\left(\lambda \right)}\left(X_{t}\right)dt+\sqrt{2}dB_{t}$. We write $Q_{\lambda }^{M}$ for the corresponding candidate distribution and $P_{\lambda }^{M}$ for the Metropolis–Hastings kernel with proposal $Q_{\lambda }^{M}$ and target $\pi^{\left(\lambda \right)}$.

We present results for the Langevin algorithm in two settings. Initially, we consider more restrictive conditions under which our upper bound on the spectral gap depends on the tail behaviour of *π* in a particularly explicit manner, and then give a broader result.

Condition 2 Assume the following:

- *π* has a density of the form $\pi \left(x\right)=\pi_{1}\left(x_{1}\right)\pi_{2:n}\left(x_{2},\ldots ,x_{d}\right)$, for some densities $\pi_{1}$ and $\pi_{2:n}$ on R and $R^{d-1}$, respectively.
- For some *q* ∈ [0, 1), it holds that
(7) $$\left|\frac{d}{dx_{1}},\;,\log,\;,\pi_{1},\left(x_{1}\right)\right|=\Theta (|x_{1}{|}^{q})\;\text{as}\;|x_{1}|↑\infty .$$

Theorem 2 *If Condition* 2 *holds, then there is a* *γ* > 0 *such that*

$$\text{Gap}\left(P_{\lambda }^{M}\right)=O\left(e^{-\gamma \lambda^{-(1+q)}\;+\;q\;\log\left(\lambda \right)}\right)\;\text{as}\;\lambda ↓0.$$

When compared with the random walk algorithm, Theorem 2 shows that the Langevin scheme is much less robust to heterogeneity. Indeed, the spectral gap decays *exponentially fast* in $\lambda^{-(1+q)}$, meaning that even small errors in the choice of step‐size can have a large impact on algorithm efficiency, and so practitioners must invest considerable effort tuning the algorithm for good performance, as shown through simulations in Sections 5 and 6. Theorem 2 also illustrates that the Langevin algorithm is more sensitive to *λ* when the tails of *π*(·) are lighter. This is intuitive, as in this setting gradient terms can become very large in certain regions of the state space.

Remark 1 Theorem 2 (and Theorem 4 below) could be extended to the case *q* ≥ 1 in Equation (7); however, in these cases, samplers typically fail to be geometrically ergodic when *λ* is small (Livingstone et al., 2019; Roberts & Tweedie, 1996) meaning the spectral gap is typically 0 and the theorem becomes trivial.

Remark 2 Condition 2(ii) could be replaced with the simpler requirement that $|\nabla \;\log\;\pi_{1}\left(x_{1}\right)|↑\infty$, with the corresponding bound $\text{Gap}\left(P_{\lambda }^{M}\right)=O\left(e^{-1/\lambda }\right)$.

A different set of conditions, which hold much more generally, and corresponding upper bound are presented below.

Condition 3 Assume the following:

- There is a *γ* > 0 such that
(8) $$\underset{|x_{1}|\to \infty }{\text{lim inf}}\left(\underset{\left(x_{2},\ldots ,x_{d}\right)\in R^{d-1}}{\inf},\left|\frac{\partial \;\log\;\pi (x)}{\partial x_{1}}\right|,{\left\|x\right\|}_{2}^{\gamma }\right)>0,$$
- Given *X* ∼ *π* there is a *β* > 0 such that
(9) $${P(\|X\|}_{2}>t)=O\left(e^{-t^{\beta }}\right)\;\text{as}\;t\to \infty .$$

Theorem 3 *If Condition* 3 *holds*, *then*

$$\text{Gap}\left(P_{\lambda }^{M}\right)=O\left(e^{-\lambda^{-\alpha }}\right)\;\mathrm{as}\;\lambda ↓0,$$

*for some* *α* > 0, *which can be taken as* *α* = min{*β*/2,*β*/*γ*,2/3}.

We expect Condition 3 to be satisfied in many commonly encountered scenarios, with the exception of particularly heavy‐tailed models. In the exponential family class $\pi \left(x\right)\propto \exp\{-\alpha \|x{\|}_{2}^{\beta }\}$, for example, Condition 3 holds for any *α* and *β* > 0 (see proof in the supplement).

#### 2.3.3 Hamiltonian Monte Carlo

In Hamiltonian Monte Carlo, we write the current point $x\in R^{d}$ as *x*(0), and construct the proposal *y* := *x*(*L*) for some prescribed integer *L* using the update

(10) $$x\left(L\right)=x\left(0\right)+\sigma^{2}\left(\frac{L}{2},\nabla ,\;,\log,\;,\pi^{\left(\lambda \right)},\left(x\left(0\right)\right),+,\sum_{j=1}^{L-1},\left(L-j\right),\nabla ,\;,\log,\;,\pi^{\left(\lambda \right)},\left(x\left(j\right)\right)\right)+L\sigma \xi \left(0\right),$$

where each *x*(*j*) is defined recursively in the same manner, and $\xi \left(0\right)∼N\left(0,I_{d}\right)$. The transition is based on numerically solving Hamilton's equations for the Hamiltonian system $H\left(x,\xi \right)=-\log\;\pi^{\left(\lambda \right)}\left(x\right)+\xi^{T}\xi /2$ for *Lσ* units of time. The decision of whether or not the proposal is accepted is taken using the acceptance probability $\min(1,\pi^{\left(\lambda \right)}\left(y\right)/\pi^{\left(\lambda \right)}\left(x\right)\times e^{-\xi {\left(L\right)}^{T}\xi \left(L\right)/2+\xi {\left(0\right)}^{T}\xi \left(0\right)/2})$, where

$$\xi \left(L\right)=\xi \left(0\right)+\frac{\sigma }{2}\left(\nabla ,\;,\log,\;,\pi^{\left(\lambda \right)},\left(x\left(0\right)\right),+,\nabla ,\;,\log,\;,\pi^{\left(\lambda \right)},\left(x\left(L\right)\right)\right)+\sigma \sum_{j=1}^{L-1}\nabla \;\log\;\pi^{\left(\lambda \right)}\left(x\left(j\right)\right).$$

A more detailed description is given in Neal (2011). We write $P_{\lambda }^{H}$ for the corresponding Metropolis–Hastings kernel with proposal mechanism as above and target $\pi^{\left(\lambda \right)}$. Here we present a heterogeneity result under Condition 2 of the previous subsection.

Theorem 4 *If Condition* 2 *holds, then there is a* *γ* > 0 *such that*

$$\text{Gap}\left(P_{\lambda }^{H}\right)=O\left(e^{-\gamma \lambda^{-(1+q)}+q\;\log\left(\lambda \right)}\right)\;\mathrm{as}\;\lambda ↓0.$$

It is no surprise that Theorem 4 is comparable to Theorem 2, since setting *L* = 1 equates the Langevin and Hamiltonian methods.

### 2.4 The large *λ* regime

In this section, we briefly discuss the *λ* ↑ ∞ regime, where *σ* is chosen to be too small for the first component of $\pi^{\left(\lambda \right)}$, arguing that all samplers under consideration behave similarly in this regime and pay a similar price for too small tuning parameters in a given direction. The intuition for this is that as *λ* ↑ ∞ the gradient‐based proposal mechanisms discussed here all tend towards that of the random walk sampler in the first coordinate. For example, if we consider one‐dimensional models, for any x∈R we can write $\nabla \;\log\;\pi^{\left(\lambda \right)}\left(x\right)=\lambda^{-1}\nabla \;\log\;\pi \left(x/\lambda \right)$, meaning as *λ* ↑ ∞ the amount of gradient information in the proposal is reduced provided *π* is suitably regular. The following result makes this intuition precise. To avoid repetitions, we state here the result for both the Langevin and the Barker proposal that we will introduce in the next section.

Proposition 2 *Fix* x∈R *and let the density* *π* *be such that* ∇ log *π* *is bounded in some neighbourhood of zero. Then the Langevin and Barker candidate kernels* $Q_{\lambda }^{M}$ *and* $Q_{\lambda }^{B}$, *defined in Equations* (6) *and* (16) *respectively*, *both satisfy*

$$\|Q_{\lambda }^{M/B}\left(x,\cdot \right)‐Q^{R}{\left(x,\cdot \right)\|}_{\mathrm{TV}}=O\left(1/\lambda \right),$$

*where* $Q^{R}$ *is the (Gaussian) random walk candidate kernel*.

The same intuition applies to the Hamiltonian case provided *L* is fixed, since each gradient term in the proposal is also *Θ*(1/*λ*). While there are many well‐known measures of distance between two distributions, we argue that total variation is an appropriate choice here, since it has an explicit focus on how much the two kernels overlap and is invariant under bijective transformations of the state space (including re‐scaling coordinates).

While the above statements provide useful heuristic arguments, in order to obtain more rigorous results one should prove that the spectral gaps decay to 0 at the same rate as *λ* ↑ ∞, which we leave to future work. We note, however, that the conjecture that the algorithms behave similarly for large values of *λ* is supported by the simulations of Section 5.1.

### 2.5 Extensions

The lower bound of Theorem 1 extends naturally to the *k* > 1 setting, becoming instead $\Theta (\lambda^{k})$, and so the rate of decay remains polynomial in *λ* for any *k*. Analogously, we expect the corresponding upper bound for gradient‐based schemes to remain exponential and become $O(e^{-k(\gamma \lambda^{-(1+q)}+q\;\log\left(\lambda \right))})$, although the details of this are left for future work. We explore examples of this nature through simulations in Section 5 and find empirically that the single component case is informative also of more general cases. Further extensions in which a different $\lambda_{i}$ is chosen in each of the *k* directions can also be considered, with each $\lambda_{i}\;↓\;0$ at a different rate. We conjecture that in this setting the $\lambda_{i}$ that decays most rapidly will dictate the behaviour of spectral gaps, though such an analysis is beyond the scope of the present work. One could consider using a mixture of the MALA/HMC and random walk kernels in an attempt to achieve both robustness to tuning and favourable scaling properties. While this may seem promising in theory, in practice we believe that it would be difficult to achieve *both* robustness to tuning *and* favourable high‐dimensional performance from such an approach. In the next section, we consider a scheme for which the two goals can be achieved simultaneously.

## 3 COMBINING ROBUSTNESS AND EFFICIENCY

The results of Section 2 show that the two gradient‐based samplers considered there are much less robust to heterogeneity than the random walk algorithm. In this section, we introduce a novel and simple to implement gradient‐based scheme that shares the superior scaling properties of the Langevin and Hamiltonian schemes, but also retains the robustness of the random walk sampler, both in terms of geometric ergodicity and robustness to tuning.

### 3.1 Locally balanced Metropolis–Hastings

Consider a continuous‐time Markov jump process on $R^{d}$ with associated generator

(11) $$Lf\left(x\right)=\int \left[f\left(y\right)-f\left(x\right)\right]g\left(\frac{\pi (y)q(y,x)}{\pi (x)q(x,y)}\right)Q\left(x,dy\right),$$

for some suitable function $f:R^{d}\to R$, where *π*(*x*) is a probability density, *Q*(*x*, *dy*) := *q*(*x*, *y*)*dy* is a transition kernel and the *balancing* function *g*  :  (0, ∞) → (0,∞) satisfies

(12) g(t)=tg(1/t).

A discrete state‐space version of this process with symmetric *Q* was introduced in Power and Goldman (2019). The dynamics of the process are such that if the current state $X_{t}=x$, the next jump will be determined by a Poisson process with intensity

(13) $$Z\left(x\right):=\int g\left(\frac{\pi (y)q(y,x)}{\pi (x)q(x,y)}\right)Q\left(x,dy\right),$$

and the next state is drawn from the kernel

$$Q^{\left(g\right)}\left(x,dy\right):=Z{\left(x\right)}^{-1}g\left(\frac{\pi (y)q(y,x)}{\pi (x)q(x,y)}\right)Q\left(x,dy\right).$$

It is straightforward to show that L is a self‐adjoint operator on the Hilbert space $L^{2}\left(\pi \right)$ using Equation (12), implying that the process is *π*‐reversible and can therefore serve as a starting point for designing MCMC algorithms.

In the ‘locally balanced’ framework for discrete state‐space Metropolis–Hastings introduced in Zanella (2020), candidate kernels are of the form

(14) $$\tilde{Q}\left(x,dy\right)=\tilde{Z}{\left(x\right)}^{-1}g\left(\frac{\pi (y)}{\pi (x)}\right)\mu_{\sigma }\left(y-x\right)dy,$$

meaning the *embedded Markov chain* of Equation (11) with the choice $Q\left(x,dy\right):=\mu_{\sigma }\left(y-x\right)dy$, where $\mu_{\sigma }\left(y-x\right):=\sigma^{-d}\mu \left(\left(y-x\right)/\sigma \right)$ for some symmetric density *μ*. It is well‐known that the invariant density of the embedded chain does not coincide with that of the process when jumps are not of constant intensity, in this case becoming proportional to *Z*(*x*)*π*(*x*), as shown in Zanella (2020). As a result a Metropolis–Hastings step is employed to correct for the discrepancy. In Power and Goldman (2019) it is suggested that as an alternative the jump process can be simulated exactly.

The challenge with employing either of these strategies on a continuous state space is that the integral (13) will typically be intractable. To overcome this issue we take two steps, and for simplicity, we first describe these on R (there are two options on $R^{d}$ for *d* > 1, which are discussed in Section 3.3). The first step is to consider a first‐order Taylor series expansion of log *π* within *g* (again with a symmetric choice of *Q*), leading to the family of processes with generator

$$Lf\left(x\right)=\int \left[f\left(y\right)-f\left(x\right)\right]g\left(e^{\nabla \;\log\;\pi (x)(y-x)}\right)\mu_{\sigma }\left(y-x\right)dy.$$

We refer to candidate kernels in Metropolis–Hastings algorithms that are constructed using the embedded Markov chain of this new process as *first‐order* locally balanced proposals, taking the form

(15) $$Q^{\left(g\right)}\left(x,dy\right)=Z{\left(x\right)}^{-1}g\left(e^{\nabla \;\log\;\pi (x)(y-x)}\right)\mu_{\sigma }\left(y-x\right)dy,$$

where $Z\left(x\right):=\int g\left(e^{\nabla \;\log\;\pi (x)(y-x)}\right)\mu_{\sigma }\left(y-x\right)dy$.

Remark 3 One can also think at Equation (12) as a requirement to ensure that the proposals in Equation (15) are exact (i.e. *π*‐reversible) at the first order. In particular, in the supplement, it is shown that a proposal $Q^{\left(g\right)}$ defined as in Equation (15) is *π*‐reversible for *π* log‐linear if and only if Equation (12) holds.

The second step is to note that, if particular choices of *g* are made, then *Z*(*x*) becomes tractable. In fact, if the balancing function $g\left(t\right)=\sqrt{t}$ and a Gaussian kernel $\mu_{\sigma }$ are chosen, then the result is the Langevin proposal

$$Q^{M}\left(x,dy\right)\propto e^{\nabla \;\log\;\pi (x)(y-x)/2}\mu_{\sigma }\left(y-x\right)dy.$$

Thus, MALA can be viewed as a particular instance of this class. Other choices of *g* are, however, also possible, and give rise to different gradient‐based algorithms. In the next section we explore what a sensible choice of *g* might look like.

### 3.2 The Barker proposal on R

The requirement for the balancing function *g* to satisfy *g*(*t*) = *tg*(1/*t*) is in fact also imposed on the acceptance rate of a Metropolis–Hastings algorithm to produce a *π*‐reversible Markov chain. Indeed, setting *t* := *π*(*y*)*q*(*y*, *x*)/(*π*(*x*)*q*(*x*, *y*)) and assuming *α*(*x*, *y*) := *α*(*t*), then the detailed balance equations can be written *α*(*t*) = *tα*(1/*t*). Possible choices of *g* can therefore be found by considering suggestions for *α* in the literature. One choice proposed in Barker (1965) is

$$g\left(t\right)=\frac{t}{1+t}.$$

The work of Peskun (1973) and Tierney (1998) showed that this choice of *α* is inferior to the more familiar Metropolis–Hasting rule *α*(*t*) = min(1, *t*) in terms of asymptotic variance. The same conclusion cannot, however, be drawn when considering the choice of balancing function *g*.

In fact, the choice *g*(*t*) = *t*/(1+*t*) was shown to minimize asymptotic variances of Markov chain estimators in some discrete settings in Zanella (2020). In addition, as shown below, this particular choice of *g* leads to a fully tractable candidate kernel that can be easily sampled from. For this reason, we focus on this choice of *g* here, and leave the question of optimality in general for future work.

Proposition 3 *If* *g*(*t*) = *t* / (1 + *t*), *then the normalising constant* *Z*(*x*) *in Equation* (15) *is* 1/2.

The resulting proposal distribution is

(16) $$Q^{B}\left(x,dy\right)=2\frac{\mu_{\sigma }\left(y-x\right)}{1+e^{-\nabla \;\log\;\pi (x)(y-x)}}dy.$$

We refer to $Q^{B}$ as the *the Barker proposal*. A simple sampling strategy to generate $y∼Q^{B}\left(x,\cdot \right)$ is given in Algorithm 1.

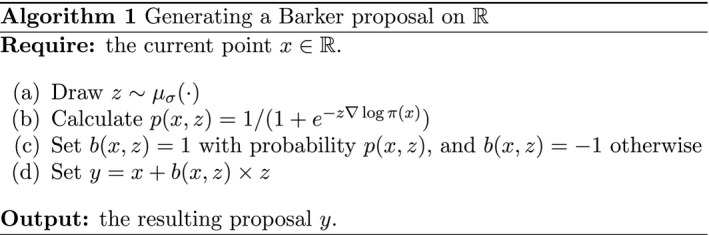

Proposition 4 *Algorithm 1 produces a sample from* $Q^{B}$ on R.

**FIGURE 1:**
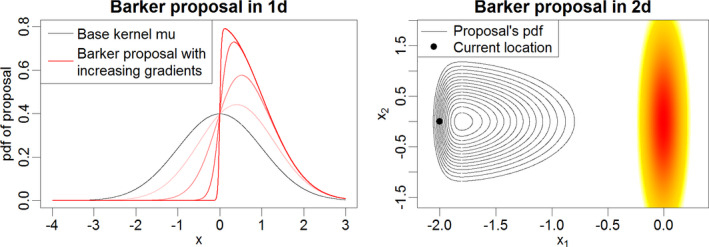
Left: density of the Barker proposal in one dimension. Current location is *x* = 0 and the four lines with increasing red intensity correspond to ∇ log *π*(*x*) equal to 1, 3, 10 and 50. Right: density of the Barker proposal in two dimensions. Solid lines display the proposal density contours, heat colours refer to the target density, and the current location is *x* = (−2, 0) [Colour figure can be viewed at wileyonlinelibrary.com]

Algorithm 1 shows that the magnitude |*y*−*x*| = |*z*| of the proposed move does not depend on the gradient ∇ log *π*(*x*) here, it is instead dictated only by the choice of symmetric kernel $\mu_{\sigma }$. The *direction* of the proposed move is, however, informed by both the magnitude and direction of the gradient. Examining the form of *p*(*x*, *z*), it becomes clear that if the signs of *z* and ∇ log *π*(*x*) are in agreement, then *p*(*x*, *z*) > 1/2, and indeed as *z*∇ log *π*(*x*) ↑ ∞ then $e^{-z\nabla \;\log\;\pi (x)}↓0$ and so *p*(*x*, *z*) ↑ 1. Hence, if the indications from ∇ log *π*(*x*) are that *π*(*x* + *z*) ≫ *π*(*x*), then it is highly likely that *b*(*x*, *z*) will be set to 1 and *y* = *x* + *z* will be the proposed move. Conversely, if *z*∇ log *π*(*x*) < 0, then there is a larger than 50% chance that the proposal will instead be *y* = *x* − *z*. As ∇ log *π*(*x*) ↑ ∞ the Barker proposal converges to $\mu_{\sigma }$ truncated on the right, and similarly to $\mu_{\sigma }$ truncated on the left as ∇ log *π*(*x*) ↓ − ∞. See Figure 1 for an illustration.

The multiplicative term $1/(1+e^{-\nabla \;\log\;\pi (x)(y-x)})$ in Equation (16), which incorporates the gradient information, injects skewness into the base kernel $\mu_{\sigma }$ (as can be clearly seen in the left‐hand plot of Figure 1). Indeed, the resulting distribution $Q^{B}$ is an example of a *skew‐symmetric* distribution (Azzalini, 2013, Eq. (1.3)). Skew‐symmetric distributions are a tractable family of (skewed) probability density functions that are obtained by multiplying a symmetric base density functionwith the cumulative distribution function (cdf) of a symmetric random variable. We refer to (Azzalini 2013 Ch. 1) for more details, including a more general version of Propositions 3 and 4. In the context of skewed distributions, the Gaussian cdf is often used, leading to the skew‐normal distribution introduced in Azzalini (1985). In our context, however, the Barker proposal (which leads to the logistic cdf *p*(*x*, *z*) in Algorithm 1) is the only skew‐symmetric distribution that can be obtained from Equation (15) using a balancing function *g* satisfying *g*(*t*) = *tg*(1/*t*). See the supplement for more detail.

### 3.3 The Barker proposal on $R^{d}$

There are two natural ways to extend the Barker proposal to $R^{d}$, for *d* > 1. The first is to treat each coordinate separately, and generate the proposal $y=(y_{1},\;\ldots ,\;y_{d})$ by applying Algorithm 1 independently to each coordinate. This corresponds to generating a $z_{i}$ and $b_{i}\left(x,z_{i}\right)$ for each *i* ∈ {1, …, *d*}, and choosing the sign of each $b_{i}$ using

$$p_{i}\left(x,z_{i}\right)=\frac{1}{1+e^{-z_{i}\partial_{i}\;\log\;\pi \left(x\right)}},$$

where $\partial_{i}\;\log\;\pi \left(x\right)$ denotes the partial derivative of log *π*(*x*) with respect to $x_{i}$. Writing $Q_{i}^{B}\left(x,dy_{i}\right)$ to denote the resulting Barker proposal candidate kernel for the *i*th coordinate, the full candidate kernel $Q^{B}$ can then be written

(17) $$Q^{B}\left(x,dy\right)=\prod_{i=1}^{d}Q_{i}^{B}\left(x,dy_{i}\right).$$

The full Metropolis–Hastings scheme using the Barker proposal mechanism for a target distribution is given in Algorithm 2 (see the supplement for more details and variations of the algorithm, such as a pre‐conditioned version). Note that the computational cost of each iteration of the algorithm is essentially equivalent to that of MALA and will be typically dominated by the cost of computing the gradient and density of the target.

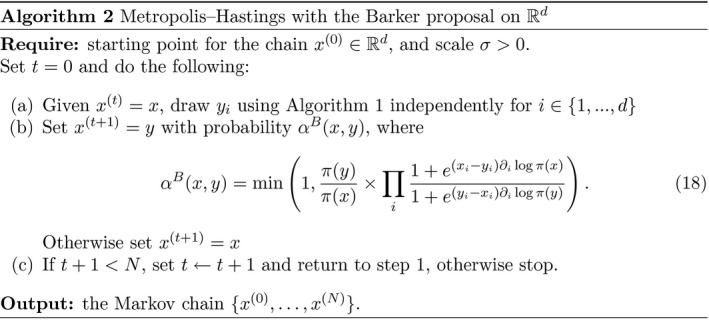

The second approach to deriving a multivariate Barker proposal consists of sampling $z\in R^{d}$ from a *d*‐dimensional symmetric distribution, and then choosing whether or not to flip the sign of *every* coordinate at the same time, using a single global $\overset{ˇ}{b}\left(x,z\right)\in \left\{-1,1\right\}$, to produce the global proposal $y=x+\overset{ˇ}{b}\left(x,z\right)\times z$. In this case, the probability that $\overset{ˇ}{b}\left(x,z\right)=1$ will be

(19) $$\overset{ˇ}{p}\left(x,z\right)=\frac{1}{1+e^{-z^{T}\nabla \;\log\;\pi \left(x\right)}}.$$

This second approach does not allow gradient information to feed into the proposal as effectively as in the first case. Specifically, only the global inner product $z^{T}\nabla \;\log\;\pi \left(x\right)$ is considered, and the decision to alter the sign of every component of *z* is taken based solely on this value. In other words, once $z∼\mu_{\sigma }$ has been sampled, gradient information is only used to make a single binary decision of choosing between the two possible proposals *x* + *z* and *x* − *z*, while in the first strategy gradient information is used to choose between $2^{d}$ possible proposals $\left\{x+b\cdot z:b\in {\left\{-1,1\right\}}^{d}\right\}$ (where $b\cdot z:=(b_{1}z_{1},\ldots ,b_{d}z_{d})$). Indeed, the following proposition shows that the second strategy cannot improve over the RWM by more than a factor of two.

Proposition 5 *Let* ${\overset{ˇ}{P}}^{B}$ *denote the modified Barker proposal on* $R^{d}$ *using Equation* (19). *Then* $\text{Gap}\left(P^{R}\right)\geq \text{Gap}\left({\overset{ˇ}{P}}^{B}\right)/2$.

One can also make a stronger statement than the above proposition, namely that if this strategy is employed, only a constant factor improvement over the RWM can be achieved in terms of asymptotic variance, for any $L^{2}\left(\pi \right)$ function of interest. Given Proposition 5 we choose to use the first strategy described to produce Barker proposals on $R^{d}$, and the multi‐dimensional candidate kernel given in Equation (17). In the following sections, we will show both theoretically and empirically that this choice does indeed have favourable robustness and efficiency properties.

## 4 ROBUSTNESS, SCALING AND ERGODICITY RESULTS FOR THE BARKER PROPOSAL

In this section, we establish results concerning robustness to tuning, scaling with dimension and geometric ergodicity for the Barker proposal scheme. As we will see, the method not only enjoys the superior efficiency of gradient‐based algorithms in terms of scaling with dimension, but also shares the favourable robustness properties of the RWM when considering both robustness to tuning and geometric ergodicity.

### 4.1 Robustness to tuning

We now examine the robustness to tuning of the Barker proposal using the framework introduced in Section 2. We write $Q_{\lambda }^{B}$ and $P_{\lambda }^{B}$ to denote the candidate and Metropolis–Hastings kernels for the Barker proposal targeting the distribution $\pi^{\left(\lambda \right)}$ defined therein, and $P^{B}$ for the case *λ* = 1. The following result characterizes the behaviour of the spectral gap of $P_{\lambda }^{B}$ as *λ* ↓ 0.

Theorem 5 *Assume Condition* 1 *and* $\text{Gap}(P^{B})>0$. *Then it holds that*

$$\text{Gap}\left(P_{\lambda }^{B}\right)=\Theta \left(\lambda \right),\;as\;\lambda ↓0.$$

Comparing Theorem 5 with Theorems 1, 2, 3, 4 from Section 2.3, we see that the Barker proposal inherits the robustness to tuning of random walk schemes and is significantly more robust than the Langevin and Hamiltonian algorithms. In the next section, we establish general conditions under which $\text{Gap}(P^{B})>0$.

### 4.2 Geometric ergodicity

In this section, we study the class of target distributions for which the Barker proposal produces a geometrically ergodic Markov chain. We show that geometric ergodicity can be obtained even when the gradient term in the proposal grows faster than linearly, which is typically not the case for MALA and HMC.

Recall that a Markov chain is called *geometrically ergodic* if

(20) $$\|P^{t}{\left(x,\cdot \right)-\pi \left(\cdot \right)\|}_{\mathrm{TV}}\leq CV\left(x\right)\rho^{t},\;t\geq 1,$$

for some *C* < ∞, Lyapunov function $V:R^{d}\to \left[1,\infty \right)$, and *ρ* < 1, where ${\left\|\mu \left(\cdot \right)-\nu \left(\cdot \right)\right\|}_{\mathrm{TV}}\;:=\;\sup_{A\in B}\;\left|\mu \left(A\right)-\nu \left(A\right)\right|$ for probability measures *μ* and *ν*. When such a condition can be established for a reversible Markov chain, then a central limit theorem exists for any square‐integrable function (Roberts & Rosenthal, 2004).

We prove geometric ergodicity results for generic proposals as in Equation (15), assuming *g* to be bounded and monotone, and $\mu_{\sigma }$ to have lighter than exponential tails. Following the discussion in Section 3.3, we consider proposals that are independent across components, leading to

(21) $$Q^{\left(g\right)}\left(x,dy\right)=\prod_{i=1}^{d}Q_{i}^{\left(g\right)}\left(x,dy_{i}\right)=\prod_{i=1}^{d}\frac{g\left(e^{\partial_{i}\;\log\;\pi \left(x\right)\left(y_{i}-x_{i}\right)}\right)\mu_{\sigma }\left(y_{i}-x_{i}\right)dy_{i}}{Z_{i}\left(x\right)},$$

where $Z_{i}\left(x\right):=\int_{R}g\left(e^{\partial_{i}\;\log\;\pi \left(x\right)\left(y_{i}-x_{i}\right)}\right)\mu_{\sigma }\left(y_{i}-x_{i}\right)dy_{i}$. With a slight abuse of notation, we use $\mu_{\sigma }$ to represent one and *d*‐dimensional densities. The Barker proposal in Equation (17) is the special case obtained by taking *g*(*t*) = *t*/(1+*t*).

For the results of this section, we make the simplifying assumption that *π* is spherically symmetric outside a ball of radius *R* < ∞.

Condition 4 There exists *R* < ∞ and a differentiable function *f* : (0,∞) → (0,∞) with $\lim_{r\to \infty }\;f^{\prime }\left(r\right)=-\infty$ and $f^{\prime }\left(r\right)$ non‐increasing for *r* > *R* such that log *π*(*x*) = *f*(‖*x*‖) for *r* > *R*.

Theorem 6 *Let* *g* : (0, ∞)→(0, ∞) *be a bounded and non‐decreasing function*, $\int_{R}\;\exp\left(sw\right)\mu_{\sigma }\left(w\right)dw<\infty$ *for every* *s* > 0, *and* ${\text{inf}}_{w\in (‐\delta ,\delta )}\mu_{\sigma }\left(w\right)>0$ *for some* *δ* > 0. *If the target density* *π* *satisfies Condition* 4, *then the Metropolis*–*Hastings chain with proposal* $Q^{\left(g\right)}$ *is* *π*‐*a.e. geometrically ergodic*.

We note that tail regularity assumptions such as Condition 4 are common in this type of analysis (e.g. Durmus et al., 2017a; Jarner & Hansen, 2000). As an intuitive example, the condition is satisfied in the exponential family $\pi \left(x\right)\propto \exp(-\alpha \|x{\|}^{\beta })$ for all *β* > 1. As a contrast, for MALA and HMC it is known that for *β* > 2 the sampler fails to be geometrically ergodic (Livingstone et al., 2019; Roberts & Tweedie, 1996). We expect the Barker proposal to be geometrically ergodic also for the case *β* = 1, although we do not prove it in this work. It is worth noting that for the MALA choice $g\left(t\right)=\sqrt{t}$ is unbounded above, which is a central reason for the lack of stability compared to bounded choices such as *g*(*t*) = *t*/(1 + *t*) employed in the Barker scheme.

### 4.3 Scaling with dimensionality

In this section, we provide preliminary results suggesting that the Barker proposal enjoys scaling behaviour analogous to that of MALA in high‐dimensional settings, meaning that under appropriate assumptions it requires the number of iterations per effective sample to grow as $\Theta (d^{1/3})$ with the number of dimensions *d* as *d* → ∞. Similarly to Section 4.2, we prove results for general proposals $Q^{\left(g\right)}$ as in Equation (21) with balancing functions *g* satisfying *g*(*t*) = *t* *g*(1/*t*). The Barker proposal is a special case of the latter family.

We perform an asymptotic analysis for *d* → ∞ using the framework introduced in Roberts et al. (1997). The main idea is to study the rate at which the proposal step size *σ* needs to decrease as *d* → ∞ to obtain well‐behaved limiting behaviour for the MCMC algorithm under consideration (such as a *Θ*(1) acceptance rate and convergence to a non‐trivial diffusion process after appropriate time re‐scaling). Based on the rate of decrease of *σ*, one can infer how the number of MCMC iterations required for each effective sample increases as *d* → ∞. For example, in the case of the RWM, $\sigma^{2}$ must be scaled as $\Theta (d^{-1})$ as *d* → ∞ to have a well‐behaved limit (Roberts et al., 1997), which leads to RWM requiring *Θ*(*d*) iterations for each effective sample. By contrast, for MALA it is sufficient to take $\sigma^{2}=\Theta \left(d^{-1/3}\right)$ as *d* → ∞, which leads to only $\Theta (d^{1/3})$ iterations for each effective sample (Roberts & Rosenthal, 1998). While these analyses are typically performed under simplifying assumptions, such as having a target distribution with i.i.d. components, the results have been extended in many ways (e.g. removing the product‐form assumption, see Mattingly et al., 2012) obtaining analogous conclusions. See also Beskos et al. (2013) for optimal scaling analysis of HMC and Roberts and Rosenthal (2016) for rigorous connections between optimal scaling results and computational complexity statements.

In this section, we focus on the scaling behaviour of Metropolis–Hastings algorithms with proposal $Q^{\left(g\right)}$ as in Equation (21), when targeting distributions of the form $\pi \left(x\right)=\prod_{i=1}^{d}f\left(x_{i}\right)$, where *f* is a one‐dimensional smooth density function. Given the structure of $Q^{\left(g\right)}$ and *π*(·), the acceptance rate takes the form $\alpha \left(x,y\right)=\min\{1,\prod_{i=1}^{d}\alpha_{i}\left(x_{i},y_{i}\right)\}$, where

(22) $$\alpha_{i}\left(x_{i},y_{i}\right)=\frac{f(y_{i})}{f(x_{i})}\frac{g(e^{\phi^{\prime }\left(y_{i}\right)\left(x_{i}-y_{i}\right)})}{g(e^{\phi^{\prime }\left(x_{i}\right)\left(y_{i}-x_{i}\right)})}\frac{Z_{i}\left(x_{i}\right)}{Z_{i}\left(y_{i}\right)},$$

and *ϕ* = log *f*. In such a context, the scaling properties of the MCMC algorithms under consideration are typically governed by the behaviour of $\log(\alpha_{i}\left(x_{i},y_{i}\right))$ as $y_{i}$ gets close to $x_{i}$, or more precisely by degree of the leading term in the Taylor series expansion of $\log(\alpha_{i}\left(x_{i},x_{i}+\sigma u_{i}\right))$ in powers of *σ* as *σ* → 0 for fixed $x_{i}$ and $u_{i}$. For example, in the case of the RWM one has $\log\left(\alpha_{i}\left(x_{i},x_{i}+\sigma u_{i}\right)\right)=\Theta \left(\sigma \right)$ as *σ* → 0, which in fact implies the proposal variance $\sigma^{2}$ must decrease at a rate $\Theta (d^{-1})$ to obtain a non‐trivial limit. By contrast, when the MALA proposal is used, one has $\log\left(\alpha_{i}\left(x_{i},x_{i}+\sigma u_{i}\right)\right)=\Theta \left(\sigma^{3}\right)$ as *σ*→0, which in turn leads to $\sigma^{2}=\Theta \left(d^{-1/3}\right)$. See Sections 2.1–2.2 of Durmus et al. (2017b) for a more detailed and rigorous discussion on the connection between the Taylor series expansion of $\log(\alpha_{i}\left(x_{i},y_{i}\right))$ and MCMC scaling results. The following proposition shows that the condition *g*(*t*) = *t* *g*(1/*t*), when combined with some smoothness assumptions, is sufficient to ensure that the proposals $Q^{\left(g\right)}$ lead to $\log\left(\alpha_{i}\left(x_{i},x_{i}+\sigma u_{i}\right)\right)=O\left(\sigma^{3}\right)$ as *σ* → 0.

Proposition 6 *Let* *g* : (0, ∞) → (0, ∞) *and* *g*(*t*) = *t* *g*(1/*t*) *for all* *t*. *If* *g* *is three times continuously differentiable and* $\int_{R}g^{\left(j\right)}\left(e^{\mathrm{sw}}\right)\mu \left(w\right)dw<\infty$ *for all* *s* > 0 *and* *j* ∈ {0, 1, 2, 3}, *where* $g^{\left(j\right)}:\left(0,\infty \right)\;\to \;\left(0,\infty \right)$ *is the* *j* *th derivative of* *g*, *then*

(23) $$\log\left(\alpha_{i}\left(x_{i},x_{i}+\sigma u_{i}\right)\right)=O\left(\sigma^{3}\right)\;\mathrm{as}\;\sigma \to 0,$$

*for any* $x_{i}$ *and* $u_{i}$ *in* R.

Proposition 6 suggests that Metropolis–Hastings algorithms with proposals $Q^{\left(g\right)}$ such that *g*(*t*) = *t* *g*(1/*t*) have scaling behaviour analogous to MALA, meaning that $\sigma^{2}=\Theta \left(d^{-1/3}\right)$ is sufficient to ensure a non‐trivial limit and thus $\Theta (d^{1/3})$ iterations are required for each effective sample. To make these arguments rigorous, one should prove weak convergence results for *d* → ∞, as in Roberts and Rosenthal (1998). Proving such a result for a general *g* would require a significant amount of technical work, thus going beyond the scope of this section. In this paper we rather support the conjecture of $\Theta (d^{1/3})$ scaling for $Q^{\left(g\right)}$ by means of simulations (see Section 5.2). While Proposition 6 only shows $\log\left(\alpha_{i}\left(x_{i},x_{i}+\sigma u_{i}\right)\right)=O\left(\sigma^{3}\right)$, it is possible to show that $\log\left(\alpha_{i}\left(x_{i},x_{i}+\sigma u_{i}\right)\right)=\Theta \left(\sigma^{3}\right)$ with some extra assumptions on *ϕ* to exclude exceptional cases (see the supplement for more detail).

## 5 SIMULATIONS WITH FIXED TUNING PARAMETERS

Throughout Sections 5 and 6, we choose the symmetric density $\mu_{\sigma }$ within the random walk and Barker proposals to be $N(0,\sigma^{2}I_{d})$ for simplicity. Note, however, that any symmetric density $\mu_{\sigma }$ could in principle be used. It would be interesting to explore the impact of different choices of $\mu_{\sigma }$ to the performances of the Barker algorithm, and we leave such a comparison to future work.

### 5.1 Illustrations of robustness to tuning

**FIGURE 2:**
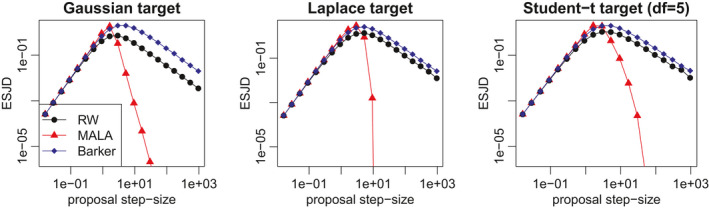
Expected squared jump distance against proposal step‐size for random walk Metropolis, Metropolis‐adjusted Langevin algorithm and Barker on different one‐dimensional targets [Colour figure can be viewed at wileyonlinelibrary.com]

We first provide an illustration of the robustness to tuning of the random walk, Langevin and Barker algorithms in three simple one‐dimensional settings. In each case we approximate the expected squared jump distance (ESJD) using $10^{4}$ Monte Carlo samples and standard Rao–Blackwellisation techniques, across of range of different proposal step‐sizes between 0.01 and 100. As is clearly shown in Figure 2, all algorithms perform similarly when the step‐size is smaller than optimal, as suggested in Section 2.4. As the step‐size increases beyond this optimum, however, behaviours begin to differ. In particular, the ESJD for MALA rapidly decays to zero, whereas in the random walk and Barker cases the reduction is much less pronounced. In fact, the rate of decay is similar for the two schemes, which is to be expected following the results of Sections 4.1 and 2.3. See the supplement for a similar illustration on a 20‐dimensional example.

### 5.2 Comparison of efficiency on isotropic targets

**FIGURE 3:**
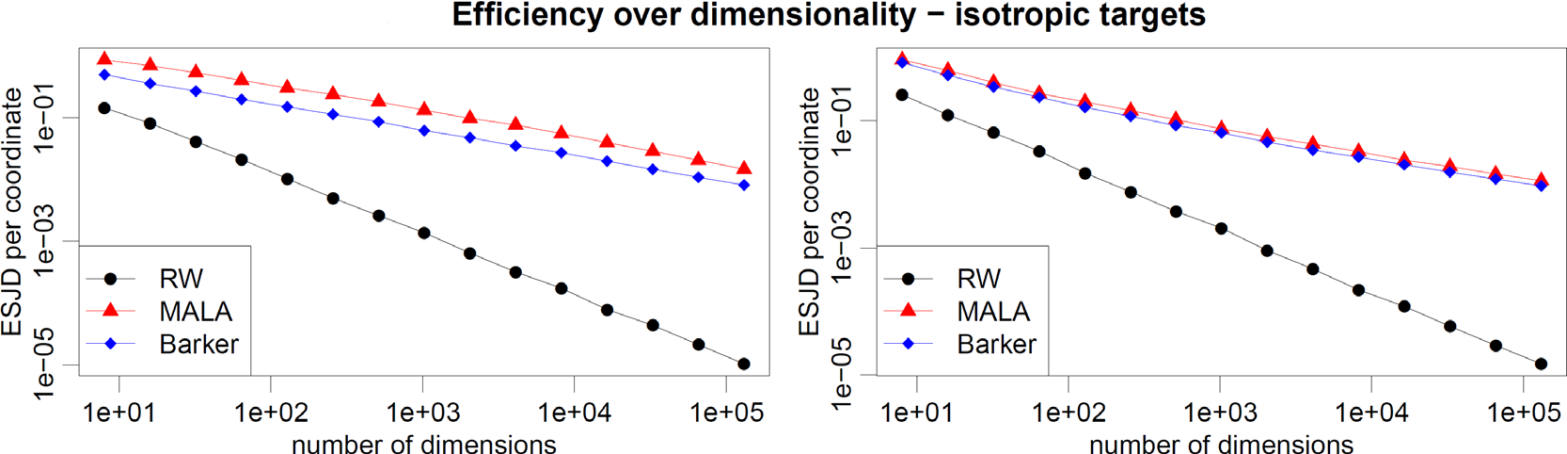
Expected squared jump distance against dimensionality for random walk Metropolis, Metropolis‐adjusted Langevin algorithm and Barker schemes with optimally‐tuned step size. The target distribution has i.i.d. coordinates following either a Gaussian distribution (left plot) or a hyperbolic one (right plot) [Colour figure can be viewed at wileyonlinelibrary.com]

Next we compare the expected squared jump distance of the random walk, Langevin and Barker schemes when sampling from isotropic distributions of increasing dimension, with optimised proposal scale (chosen to maximise expected squared jumping distance). This set‐up is favourable to MALA, which is the least robust scheme among the three, as the target distribution is homogeneous and the proposal step‐size optimally chosen. We consider target distributions with independent and identically distributed (i.i.d.) components, corresponding to the scenario studied theoretically in Section 4.3. We set the distribution of each coordinate to be either a standard normal distribution or a hyperbolic distribution, corresponding to $\log\;\pi \left(x\right)=-\sum_{i=1}^{d}x_{i}^{2}/2+\text{cons}t$ and $\log\;\pi \left(x\right)=-\sum_{i=1}^{d}{\left(0.1+x_{i}^{2}\right)}^{1/2}+\text{const}$, respectively. Figure 3 shows how the ESJD per coordinate decays as dimension increases for the three algorithms. For MALA and Barker, the ESJD appears to decrease at the same rate as *d* increases, which is in accordance with the preliminary results in Section 4.3. In the Gaussian case, MALA outperforms Barker roughly by a factor of 2 regardless of dimension (more precisely, the ESJD ratio lies between 1.7 and 2.5 for all values of *d* in Figure 3), while in the hyperbolic case the same factor is around 1.2, again independently of dimension (ESJD ratio between 1.1 and 1.25 for all values of *d* in Figure 3). The rate of decay for the RWM is faster, as predicted by the theory.

## 6 SIMULATIONS WITH ADAPTIVE MARKOV CHAIN MONTE CARLO

In this section, we illustrate how robustness to tuning affects the performance of adaptive MCMC methods.

### 6.1 Adaptation strategy and algorithmic set‐up

We use Algorithm 4 in Section 5 of Andrieu and Thoms (2008) to adapt the tuning parameters within each scheme. Specifically, in each case, a Markov chain is initialised using a chosen global proposal scale $\sigma_{0}$ and an identity pre‐conditioning matrix $\Sigma_{0}=I_{d}$, and at each iteration the global scale and pre‐conditioning matrix are updated using the equations

(24) $$\log\left(\sigma_{t}\right)=\log\left(\sigma_{t-1}\right)+\gamma_{t}\times \left(\alpha \left(X^{\left(t\right)},Y^{\left(t\right)}\right)-{\bar{\alpha }}_{*}\right)$$

(25) $$\mu_{t}=\mu_{t-1}+\gamma_{t}\times \left(X^{\left(t\right)}-\mu_{t-1}\right)$$

(26) $$\Sigma_{t}=\Sigma_{t-1}+\gamma_{t}\times \left(\left(X^{\left(t\right)}-\mu_{t}\right){\left(X^{\left(t\right)}-\mu_{t}\right)}^{T}-\Sigma_{t-1}\right).$$

Here $X^{\left(t\right)}$ denotes the current point in the Markov chain, $Y^{\left(t\right)}$ is the proposed move, $\mu_{0}=0$, ${\bar{\alpha }}_{*}$ denotes some ideal acceptance rate for the algorithm and the parameter $\gamma_{t}$ is known as the learning rate. We set ${\bar{\alpha }}_{*}$ to be 0.23 for RWM, 0.57 for MALA and 0.40 for Barker. We tried changing the value of ${\bar{\alpha }}_{*}$ for Barker in the range [0.2, 0.6] without observing major differences. In our simulations, we constrain $\Sigma_{t}$ to be diagonal (i.e., all off‐diagonal terms in Equation (26) are set to 0). This is often done in practice to avoid having to learn a dense pre‐conditioning matrix, which has both a high computational cost and would require a large number of MCMC samples. See the supplement for full details on the pre‐conditioned Barker schemes obtained with both diagonal and dense matrix $\Sigma_{t}$, including pseudo‐code of the resulting algorithms.

We set the learning rate to $\gamma_{t}:=t^{-\kappa }$ with *κ* ∈ (0.5, 1), as for example suggested in (Shaby & Wells, 2010). Small values of *κ* correspond to more aggressive adaptation, and for example Shaby and Wells (2010) suggest using *κ* = 0.8. In the simulations of Section 6.2, we use *κ* = 0.6 as this turned out to be a good balance between fast adaptation and stability for MALA (*κ* = 0.8 resulted in too slow adaptation, while values of *κ* lower than 0.6 led to instability). The adaptation of RWM and Barker was not very sensitive to the value of *κ*. Unless specified otherwise, all algorithms are randomly initialized with each coordinate sampled independently from a normal distribution with standard deviation 10. Following the results from the optimal scaling theory (Roberts & Rosenthal, 2001), we set the starting value for the global scale as $\sigma_{0}^{2}=2.4^{2}/d$ for RWM and $\sigma_{0}^{2}=2.4^{2}/d^{1/3}$ for MALA. For Barker we initialize $\sigma_{0}$ to the same values as MALA.

### 6.2 Performance on target distributions with heterogeneous scales

In this section, we compare the adaptive algorithms described above when sampling from target distributions with significant heterogeneity of scales across their components. We consider 100‐dimensional target distributions with different types of heterogeneity, tail behaviour and degree of skewness according to the following four scenarios:

1. (*One coordinate with small scale; Gaussian target*) In the first scenario, we consider a Gaussian target with zero mean and diagonal covariance matrix. We set the standard deviation of the first coordinate to 0.01 and that of the other coordinates to 1. This scenario mirrors the theoretical framework of Sections 2 and 4.1 in which a single coordinate is the source of heterogeneity.
2. (*Coordinates with random scales; Gaussian target*) Here we modify scenario 1 by generating the standard deviations of each coordinate randomly, sampling them independently from a log‐normal distribution. More precisely, we sample $\log\left(\eta_{i}\right)∼N\left(0,1\right)$ independently for *i* = 1,…, 100, where $\eta_{i}$ is the standard deviation of the *i*th component.
3. (*Coordinates with random scales; Hyperbolic target*) In the third scenario, we change the tail behaviour of the target distribution, replacing the Gaussian with a hyperbolic distribution (a smoothed version of the Laplace distribution to ensure $\log\pi \in C^{1}\left(R^{d}\right)$). In particular, we set $\log\;\pi \left(x\right)=-\sum_{i=1}^{d}{\left(\epsilon +{\left(x_{i}/\eta_{i}\right)}^{2}\right)}^{1/2}+c$, with *ε* = 0.1 and *c* being a normalizing constant. The scale parameters ${\left(\eta_{i}\right)}_{i}$ are generated randomly as in scenario 2.
4. (*Coordinates with random scales; Skew‐normal target*) Finally, we consider a non‐symmetric target distribution, which represents a more challenging and realistic situation. We assume that the *i*th coordinate follows a skew‐normal distribution with scale $\eta_{i}$ and skewness parameter *α*, meaning that $\log\;\pi \left(x\right)=-\frac{1}{2}\sum_{i=1}^{d}{\left(x_{i}/\eta_{i}\right)}^{2}+\sum_{i=1}^{d}\;\log\;\Phi \left(\alpha x_{i}/\eta_{i}\right)+c$, with *c* being a normalizing constant. We set *α* = 4 and generate the $\eta_{i}$'s randomly as in scenario 2.

**FIGURE 4:**
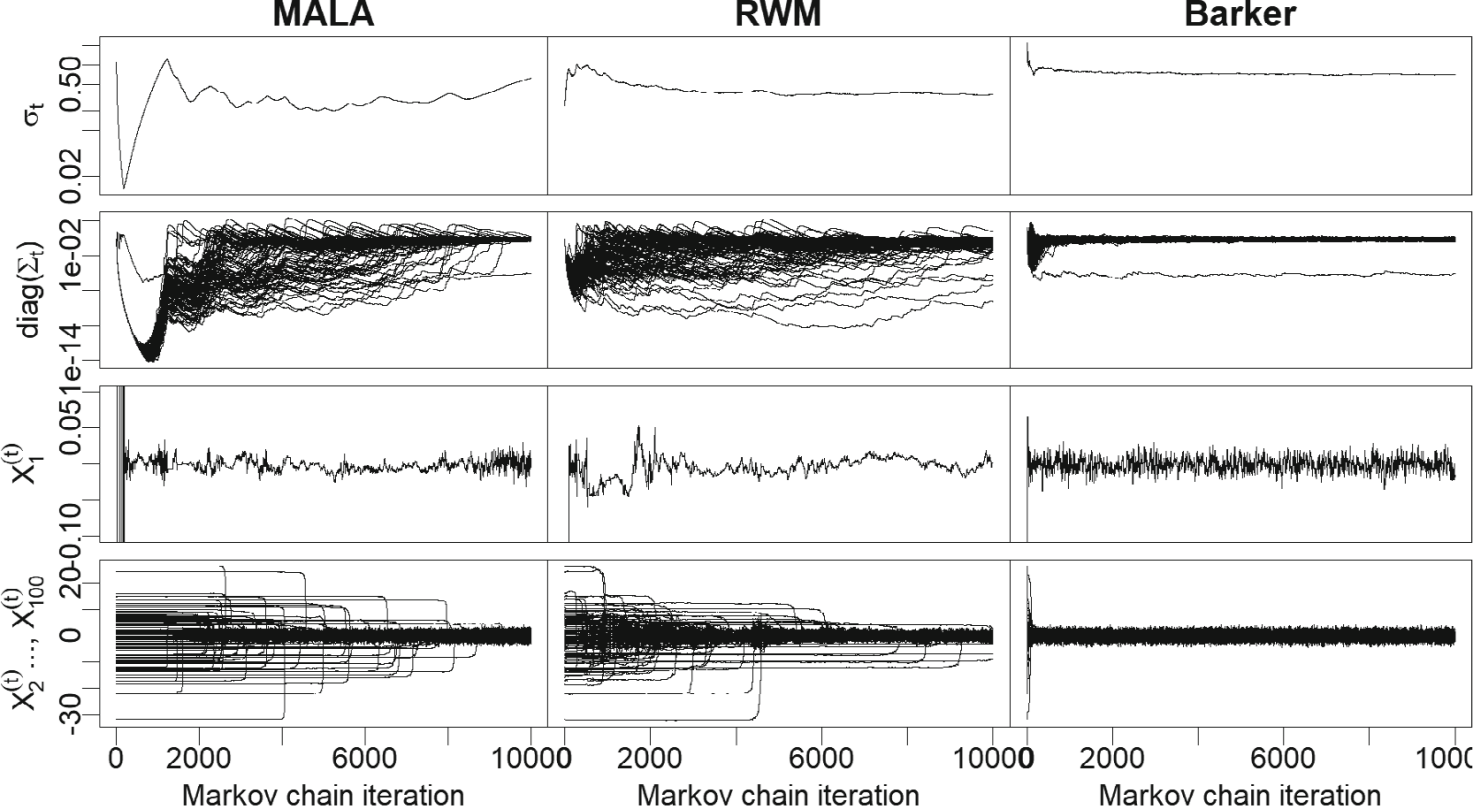
Random walk Metropolis, Metropolis‐adjusted Langevin algorithm and Barker schemes with adaptive tuning as in Equations (24), (25), (26) and learning rate set to $\gamma_{t}=t^{-\kappa }$ with *κ* = 0.6. The target distribution is a 100‐dimensional Gaussian in which the first component has standard deviation 0.01 and all others have unit scale. First row: adaptation of the global scale $\sigma_{t}$; second row: adaptation of the local scales $\text{diag}\left(\Sigma_{t}\right)={\left(\Sigma_{t,ii}\right)}_{i=1}^{100}$; third row: trace plot of first coordinate; fourth row: trace plots of coordinates from 2 to 100 (superposed)

First we provide an illustration of the behaviour of the three algorithms by plotting the trace plots of tuning parameters and MCMC trajectories—see Figure 4 for the results in scenario 1. The adaptation of tuning parameters for the Barker scheme stabilises within a few hundred iterations, after which the algorithm performance appears to be stable and efficient. On the contrary both RWM and MALA struggle to learn the heterogeneous scales and the adaptation process has either just stabilized or not yet stabilized after $10^{4}$ iterations. Looking at the behaviour of MALA in Figure 4 we see that, in order for the algorithm to achieve a non‐zero acceptance rate, the global scale parameter $\sigma_{t}$ must first be reduced considerably to accommodate the smallest scale of *π*(·). At this point the algorithm can slowly begin to learn the components of the pre‐conditioning matrix $\Sigma_{t}$, but this learning occurs very slowly because the comparatively small value for $\sigma_{t}$ results in poor mixing across all other dimensions than the first. Analogous plots for scenarios 2, 3 and 4 are given in the supplement and display comparable behaviour.

We then compare algorithms in a more quantitative way, by looking at the average mean squared error (MSE) of MCMC estimators of the first moment of each coordinate, which is a standard metric in MCMC. For any $h:R^{d}\to R$, define the corresponding MSE as $E[{\left({\hat{h}}^{\left(t\right)}-E_{\pi }\left[h\right]\right)}^{2}]$ where ${\hat{h}}^{\left(t\right)}={\left(t-t_{\mathrm{burn}}\right)}^{-1}\sum_{i=t_{\mathrm{burn}}+1}^{t}h\left(X^{\left(i\right)}\right)$ is the MCMC estimator of $E_{\pi }\left[h\right]$ after *t* iterations of the algorithm. Here $t_{\mathrm{burn}}$ is a burn‐in period, which we set to $t_{\mathrm{burn}}=\left\lfloor t/2\right\rfloor$, where ⌊·⌋ denotes the floor function. Below, we report the average MSE for the collection of test functions given by $h\left(x\right)=x_{i}/\eta_{i}$ for *i* = 1, …, *d* after *t* MCMC iterations (rescaling by $\eta_{i}$ is done to give equal importance to each coordinate).

In addition, we also monitor the rate at which the pre‐conditioning matrix $\Sigma_{t}$ converges to the covariance of *π*, denoted as Σ, in order to measure how quickly the adaptation mechanism learns suitable local tuning parameters. We consider the $l^{2}$‐distance between the diagonal elements of $\Sigma_{t}$ and Σ on the log scale. This leads to the following measure of convergence of the tuning parameters after *t* MCMC iterations:

(27) $$d_{t}=E\left[\frac{1}{\sqrt{d}},{\left(\sum_{i=1}^{d},{\left(\log\left(\Sigma_{t,ii}\right)-\log\left(\Sigma_{\mathrm{ii}}\right)\right)}^{2}\right)}^{1/2}\right],$$

where the expectation is with respect the Markov chain ${\left(X^{\left(t\right)}\right)}_{t\geq 1}$. We use the log scale as it is arguably more appropriate than the natural one when comparing step‐size parameters, and we focus on diagonal terms as both $\Sigma_{t}$ and Σ are diagonal here. Monitoring the convergence of $d_{t}$ to 0 we can compare the speed at which good tuning parameters are found during the adaptation process for different schemes.

**FIGURE 5:**
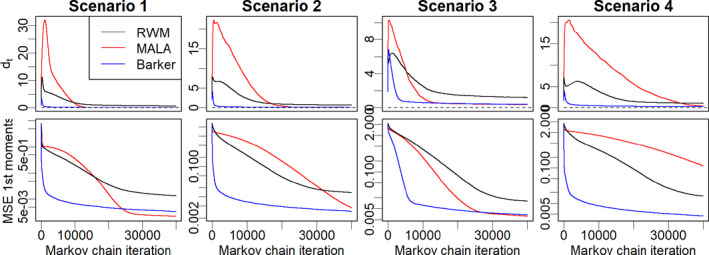
Comparison of random walk Metropolis, Metropolis‐adjusted Langevin algorithm and Barker on the four target distributions (scenarios 1 to 4) described in Section 6.2, averaging over ten repetitions of each algorithm. First row: convergence of tuning parameters, measured by $d_{t}$ defined in Equation (27). Second row: Mean Square Error of Markov chain Monte Carlo estimators of first moments averaged over all coordinates [Colour figure can be viewed at wileyonlinelibrary.com]

Figure 5 displays the evolution of $d_{t}$ and the MSE defined above over $4\;\times \;10^{4}$ iterations of each algorithms, where $d_{t}$ and the MSE are estimated by averaging over 100 independent runs of each algorithm. The results are in accordance with the illustration in Figure 4, and suggest that the Barker scheme is robust to different types of targets and heterogeneity and results in very fast adaptation, while both MALA and RWM require significantly more iterations to find good tuning parameters. The tuning parameters of MALA appear to exhibit more unstable behaviour than RWM in the first few thousands iterations (larger $d_{t}$), while after that they converge more quickly, which again is in accordance with the behaviour observed in Figure 5 and with the theoretical considerations of Sections 2 and 4.1. To further quantify the tuning period, we define the time to reach a stable level of tuning as $\tau_{\mathrm{adapt}}\left(\epsilon \right)=\inf\left\{t\geq 1:d_{t}\leq \epsilon \right\}$ for some *ε* > 0. We take *ε*=1 and report the resulting values in Table 1, denoting $\tau_{\mathrm{adapt}}\left(1\right)$ simply as $\tau_{\mathrm{adapt}}$. The results show that in these examples Barker always has the smallest adaptation time, with a speed‐up compared to RWM of at least 34x in all four scenarios, and a speed‐up compared to MALA ranging between 3x (scenario 3) and 30x (scenario 2). The adaptation times $\tau_{\mathrm{adapt}}$ tend to increase from scenarios 1 to 4, suggesting that the target distribution becomes more challenging as we move from scenarios 1 to 4. The hardest case for Barker seems to be the hyperbolic target, although even there the tuning stabilized in roughly 3000 iterations, while the hardest case for MALA is the skew‐normal, in which tuning stabilized in roughly 30,000 iterations.

**TABLE 1:** Adaptation times ($\tau_{\mathrm{adapt}}$) and mean squared errors (*MSE*) from 10 k, 20 k and 40 k iterations of the random walk Metropolis (RWM), Metropolis‐adjusted Langevin algorithm (MALA) and Barker algorithms under each of the four heterogeneous scenarios described in Section 6.2

	Method	$\tau_{\mathrm{adapt}}$	$MSE_{10k}$	$MSE_{20k}$	$MSE_{40k}$
*1*	RWM	18,757	0.200	0.036	0.013
MALA	10,785	0.348	0.016	0.002
Barker	524	0.007	0.005	0.003
*2*	RWM	19,163	0.228	0.045	0.013
MALA	17,298	0.644	0.147	0.004
Barker	542	0.007	0.005	0.003
*3*	RWM	>40 k	0.409	0.080	0.016
MALA	10,630	0.248	0.019	0.006
Barker	3294	0.012	0.009	0.007
*4*	RWM	>40 k	0.315	0.092	0.016
MALA	34,340	0.813	0.488	0.112
Barker	1427	0.008	0.006	0.004

The differences in the adaptation times have a direct implication on the resulting MSE of MCMC estimators, which is intuitive because the Markov chain will typically start sampling efficiently from *π* only once good tuning parameters are found. As we see from the second row of Figure 5 and the second part of Table 1, the MSE of Barker is already quite low (between 0.007 and 0.012) after $10^{4}$ iterations in all scenarios, while RWM and MALA need significantly more iterations to achieve the same MSE. After finding good tuning parameters and having sampled enough, MALA is slightly more efficient than Barker for the Gaussian target in scenario 1 and equally efficient in the hyperbolic target of scenario 3, which is consistent with the simulations of Section 5.2 under optimal tuning.

### 6.3 Comparison on a Poisson random effects model

In this section, we consider a Poisson hierarchical model of the form

(28) $$\begin{array}{cc} & y_{\mathrm{ij}}|\eta_{i}\;\overset{\mathrm{ind}}{∼}\;\text{Poisson}\left(\exp\left(\eta_{i}\right)\right)\;j=1,\ldots ,n_{i},\\ & \;\eta_{i}|\mu \;\overset{\mathrm{ind}}{∼}\;N\left(\mu ,\sigma_{\eta }^{2}\right)\;i=1,\ldots ,I,\\ & \;\mu ∼N(0,10^{2}), \end{array}$$

and test the algorithms on the task of sampling from the resulting posterior distribution $p(\mu ,\eta_{1},\ldots ,\eta_{I}|y)$, where $y={\left(y_{\mathrm{ij}}\right)}_{\mathrm{ij}}$ denotes the observed data. In our simulations, we set *I* = 50 and $n_{i}=5$ for all *i*, leading to 51 unknown parameters and 250 observations.

The model in Equation (28) is an example of a generalized linear model that induces a posterior distribution with light tails and potentially large gradients of log *π*, which creates a challenge for gradient‐based algorithms. In particular, the task of sampling from the posterior becomes harder when either the observations ${\left(y_{\mathrm{ij}}\right)}_{\mathrm{ij}}$ contain large values or they are heterogeneous across values of *i* ∈ {1, …, *I*}. The former case results in a more peaked posterior distribution with larger gradients, while the latter induces heterogeneity across the posterior distributions of the parameters $\eta_{i}$.

In our simulations, we consider three scenarios, corresponding to increasingly challenging target distributions:

1. In the first scenario, we take $\sigma_{\eta }=1$ and generate the data **y** from the model in Equation (28) assuming the data‐generating value of *μ* to be $\mu^{*}=5$ and sampling the data‐generating values of $\eta_{1},\ldots ,\eta_{I}$ from their prior distribution.
2. In the second scenario, we increase the value of $\sigma_{\eta }$ to 3, which induces more heterogeneity across the parameters $\eta_{1},\ldots ,\eta_{I}$.
3. In the third scenario, we keep $\sigma_{\eta }=3$ and increase the values of $\mu^{*}$ to 10, thus inducing larger gradients.

**FIGURE 6:**
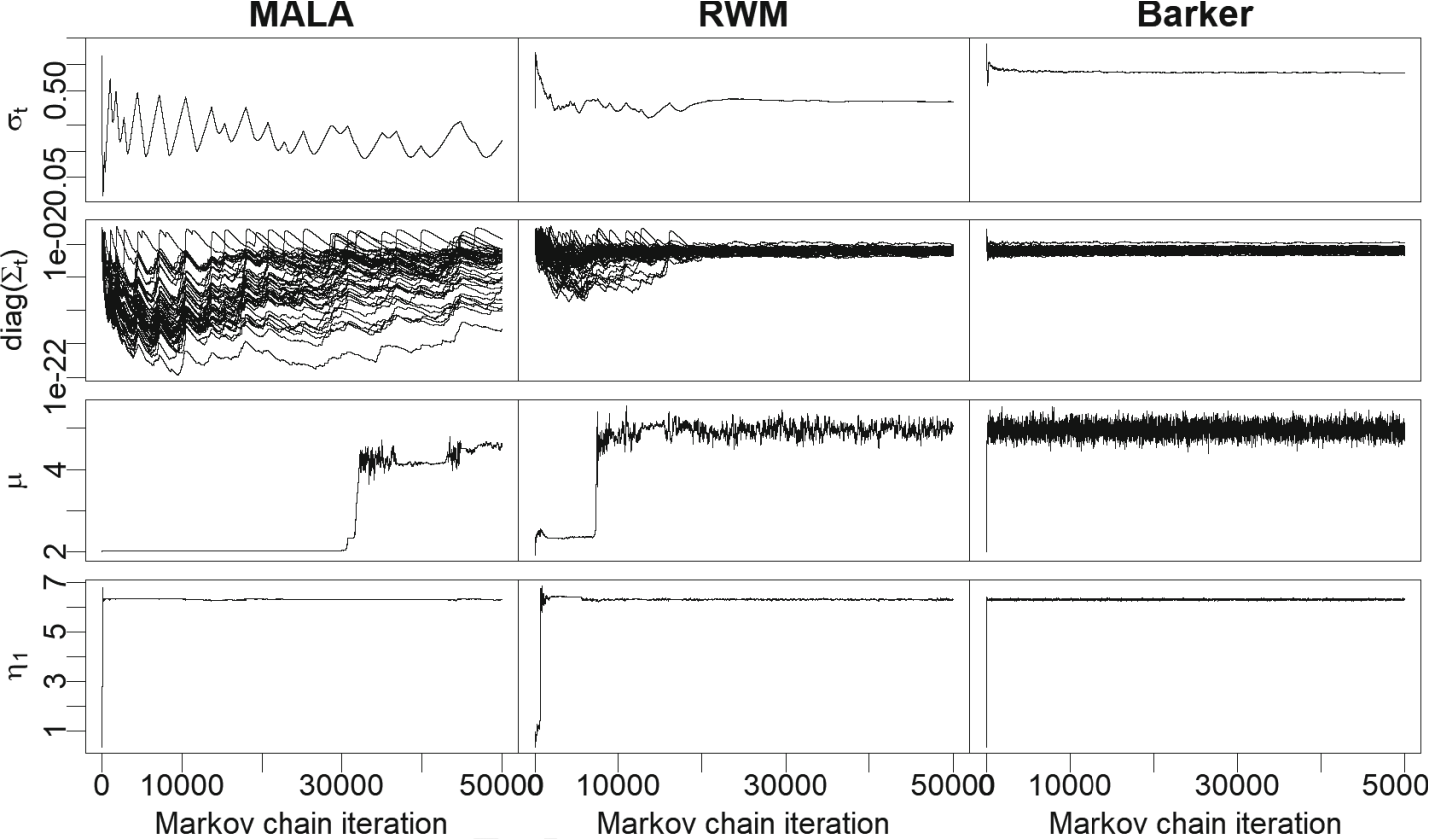
Behaviour of random walk Metropolis, Metropolis‐adjusted Langevin algorithm and Barker on the posterior distribution from the Poisson hierarchical model in Equation (28). Data are generated as in the first scenario of Section 6.3. First row: adaptation of the global scale $\sigma_{t}$; second row: adaptation of the local scales $\text{diag}\left(\Sigma_{t}\right)={\left(\Sigma_{t,ii}\right)}_{i=1}^{100}$; third row: trace plot of the parameter *μ*; fourth row: trace plots of the parameter $\eta_{1}$

In each scenario, we run the algorithm directly on the joint parameter space $\left(\mu ,\eta_{1},\ldots ,\eta_{I}\right)$. Similarly to Section 6.2, we first provide an illustration of the behaviour of the tuning parameters and MCMC trace plots for RWM, MALA and Barker in Figure 6. Here all algorithms are run for $5\;\times \;10^{4}$ iterations, with the target defined in the first scenario. We use the adaptation strategy of Section 6.2 for tuning, following Equations (24), (25), (26) with *κ* = 0.6 and $\Sigma_{t}$ constrained to be diagonal, and initialize the chains from a random configuration sampled from the prior distribution of the model. In this example, the random walk converges to stationarity in roughly 10,000 iterations while the Barker scheme takes a few hundreds. By contrast MALA struggles to converge and exhibits unstable behaviour even after $5\;\times \;10^{4}$ iterations. Note that the first $3\;\times \;10^{4}$ iterations of MALA, in which the parameter *μ* appears to be constant, do not correspond to rejections but rather to moves with very small increments in the *μ* component.

We then provide a more systematic comparison between the algorithms under consideration in Table 2. In addition to RWM, MALA and Barker, we also consider a state‐of‐the‐art implementation of adaptive HMC, namely the Stan (Stan Development Team, 2020) implementation of the No‐U‐Turn Sampler (NUTS) (Hoffman & Gelman, 2014) as well as of standard HMC (referred to as ‘static HMC’ in the Stan package). The NUTS algorithm is a variant of standard HMC in which the number of leapfrog iterations, that is, the parameter *L* in Equation (10), is allowed to depend on the current state (using a ‘No‐U‐Turn’ criterion). The resulting number of leapfrog steps (and thus log‐posterior gradient evaluations) per iteration is not fixed in advance but rather tuned adaptively depending on the hardness of the problem. This is also the case for the static HMC algorithm implementation in Stan, as in that case the total integration time in Equation (10) is fixed and the step‐size and mass matrix are adapted. For both algorithms, we use the default Stan version that learns a diagonal covariance/mass matrix during the adaptation process. This is analogous to constraining the preconditioning matrix $\Sigma_{t}$ for RWM, MALA and Barker to be diagonal, as we are doing here.

**TABLE 2:** Comparison of sampling schemes on the posterior distribution arising from the Poisson hierarchical model in Equation (28)

	Method	Iterations (*n*)	Leapfrog steps/*n*	Gradient calls (*g*)	ESS	*ESS*/*g* × 100
*1*	RWM	$5\;\times \;10^{4}$	–	–	(49, 66)	–
MALA	$5\;\times \;10^{4}$	–	$5\;\times \;10^{4}$	(648, 727)	1.30 ± 2.73
Barker	$5\;\times \;10^{4}$	–	$5\;\times \;10^{4}$	(1445, 1587)	2.89 ± 0.07
HMC	$2\;\times \;10^{3}$	89.5	$1.8\;\times \;10^{5}$	(285, 1954)	0.25 ± 0.78
NUTS	$2\;\times \;10^{3}$	8.5	$1.7\;\times \;10^{4}$	(1175, 1822)	6.95 ± 1.68
*2*	RWM	$5\;\times \;10^{4}$	–	–	(0.4, 10.6)	–
MALA	$5\;\times \;10^{4}$	–	$5\;\times \;10^{4}$	(0.0, 8.0)	< 0.01
Barker	$5\;\times \;10^{4}$	–	$5\;\times \;10^{4}$	(1365, 1563)	2.73 ± 0.13
HMC	$2\;\times \;10^{3}$	797	$1.6\;\times \;10^{6}$	(25, 1949)	< 0.01
NUTS	$2\times 10^{3}$	57.7	$1.2\;\times \;10^{5}$	(942, 1826)	1.19 ± 1.14
*3*	RWM	$5\;\times \;10^{4}$	–	–	(0.0, 5.3)	–
MALA	$5\;\times \;10^{4}$	–	$5\;\times \;10^{4}$	(0.0, 0.2)	< 0.01
Barker	$5\;\times \;10^{4}$	–	$5\;\times \;10^{4}$	(1301, 1594)	2.60 ± 0.92
HMC	$2\;\times \;10^{3}$	8103	$1.6\;\times \;10^{7}$	(3.3, 899)	< 0.01
NUTS	$2\;\times \;10^{3}$	179	$3.5\;\times \;10^{5}$	(137, 348)	0.012 ± 0.14

Blocks of rows from 1 to 3 refer to the three data‐generating scenarios described in Section 6.3. All numbers are averaged across ten repetitions of each algorithm. For each algorithm we report: number of iterations; number of leapfrog steps per iteration and total number of gradient evaluations (when applicable); estimated effective sample size (ESS) (minimum and median across parameters); minimum ESS per hundred gradient evaluations (with standard deviation across the ten repetitions).

Table 2 reports the results of the simulations for the five algorithms in each of the three scenarios. For each algorithm, we report the number of log‐posterior gradient evaluations and the minimum and median effective sample size (ESS) across the 51 unknown parameters. The ESS values are computed with the effectiveSize function from the coda R package (Plummer et al., 2006), discarding the first half of the samples as burn‐in. The RWM, MALA and Barker schemes are run for $5\;\times \;10^{4}$ iterations, and the HMC and NUTS schemes for $2\;\times \;10^{3}$ iterations. The latter is the default value in the Stan package and in this example corresponds to a number of gradient evaluations between $1.7\;\times \;10^{4}$ and $1.6\;\times \;10^{7}$. All numbers in Table 2 are averaged over ten independent replications of each algorithm. We use the minimum ESS per gradient evaluation as an efficiency metric, of which we report the mean and standard deviation across the 10 replicates (multiplied by 100 to facilitate readability).

According to Table 2, NUTS is the most efficient scheme in scenario 1, while Barker is the most efficient one in scenarios 2 and 3. This is in accordance with the intuition of Barker being a more robust scheme, as the target distribution becomes more challenging as we move from scenarios 1 to 3. MALA struggles to converge to stationarity in scenarios 2 and 3 (with an estimated ESS around zero), while it performs better in scenario 1, although with a high variability across different runs (shown by the large standard deviation in the last column). The RWM displays low ESS values for all three scenarios, although with a less dramatic deterioration going from scenarios 1 to 3. Interestingly, the performances of Barker are remarkably stable across scenarios (with an ESS of around 1400), as well as across parameters for which ESS is computed (in all cases the minimum and median ESS are close to each other) and across repetitions (shown by the relatively small standard deviation in the last column). We note that NUTS is also remarkably effective taking into consideration that it is not an algorithm designed with a major emphasis on robustness, but that performance does degrade when moving from scenarios 1 to 3. As in the MALA case, static HMC struggles to converge in scenarios 2 and 3 and is not very efficient in scenario 1. Note that NUTS, and in particular HMC, compensate for the increasing difficulty of the target by increasing the number of leapfrog steps per iteration. For example, the drop in efficiency of NUTS between scenarios 1 and 2 is mostly due to the increase in average number of leapfrog iterations from 8.5 to 57.7 rather than in a decrease in ESS. Somewhat surprisingly, in static HMC the number of leapfrog steps per iteration is increased significantly more than NUTS, which could either be due to genuine algorithmic differences or to variations in the details of the adaptation strategy implemented in Stan. Overall, Barker and NUTS are the two most efficient algorithms in these simulation, with a relative efficiency that depends on the scenario under consideration: NUTS being roughly 2.4 times more efficient in scenario 1, Barker 2.3 times more efficient in scenario 2 and Barker 40 times more efficient in scenario 3.

### 6.4 Additional simulations reported in the supplement

In the supplement, we report additional simulations for some of the above experiments. As a sensitivity check, we also performed simulations using the tamed Metropolis‐adjusted Langevin algorithm (Brosse et al., 2018) and the truncated Metropolis‐adjusted Langevin algorithm (Atchade, 2006; Roberts & Tweedie, 1996), two more robust modifications to MALA in which large gradients are controlled by monitoring the size of ‖∇ log *π*(*x*)‖. The schemes do offer some added stability compared to MALA in terms of controlling large gradients, but ultimately are still very sensitive to heterogeneity of the target distribution and to the choice of the truncation level, and do not exhibit the same robustness observed in the case of the Barker scheme. See the supplement for implementation details, results and further discussion.

## 7 DISCUSSION

We have introduced a new gradient‐based MCMC method, *the Barker proposal*, and have demonstrated both analytically and numerically that it shares the favourable scaling properties of other gradient‐based approaches, along with an increased level of robustness, both in terms of geometric ergodicity and robustness to tuning (as defined in the present paper). The most striking benefit of the method appears to be in the context of adaptive MCMC. Evidence suggests that combining the efficiency of a gradient‐based proposal mechanism with a method that exhibits robustness to tuning gives a combination of stability and speed that is very desirable in this setting, and can lead to efficient sampling that requires minimal practitioner input.

The theoretical results in this paper could be extended by studying in greater depth the large *λ* regime (Section 2.4) and the high‐dimensional scaling of the Barker proposal (Section 4.3). Of course, there are many other algorithms that could be considered under the robustness to tuning framework, and it is worthwhile future work to explore which features of a scheme result in either robustness to tuning or a lack of it. Extensions to the Barker proposal that incorporate momentum and exhibit the $d^{-1/4}$ decay in efficiency with dimension enjoyed by HMC may be possible, as well as the development of other methods within the first‐order locally balanced proposal framework introduced in Section 3, or indeed schemes that are exact at higher orders.
