# Supplementary material for: The Barker proposal: Combining robustness and efficiency in gradient‐based MCMC
Source: J R Stat Soc Series B Stat Methodol. 2022 Jan 11;84(2):496–523. doi: 10.1111/rssb.12482 (PMC9303935; doi:10.1111/rssb.12482)
Supplement: Supplementary file 1 [file RSSB-84-496-s001.pdf]

Supplement for “The Barker proposal: combining robustness and efficiency in gradient-based MCMC”

Samuel Livingstone

Department of Statistical Science, University College, Gower Street, London, UK.

Giacomo Zanella

Department of Decision Sciences, BIDSA and IGIER, Bocconi University, Via Roentgen 1, Milan, Italy.

Summary. The supplementary material contains proofs of the results in Livingstone and Zanella (2020) and additional figures related to the simulations. It also includes some background on the key techniques used for the proofs of Section 2, a proof of Condition 3 for the exponential family class and details related to skew-symmetry and pre-conditioning of the Barker proposal. In this supplement, we number equations, figures and lemmas differently from the main paper, e.g. (1) rather than (1.1), to avoid confusion between the two documents.

1. Tools to bound spectral gaps

To establish lower bounds on spectral gaps we use the following Lemma.

LEMMA 1.1. *Consider two Metropolis–Hastings kernels P_1 and P_2 with associated candidate kernels $Q_1(x, dy) = q_1(x, y)dy$ and $Q_2(x, dy) = q_2(x, y)dy$ and common target distribution π. If there is a $\gamma > 0$ such that $q_1(x, y) \geq \gamma q_2(x, y)$ for all fixed x, y with $x \neq y$, then*

$$\mathrm{Gap}(P_1) \geq \gamma \mathrm{Gap}(P_2). \tag{1.1}$$

PROOF. For any $f \in L^2_{0,1}(\pi)$, it holds that

$$\begin{aligned}
&\int \{f(y) - f(x)\}^2 \pi(dx) P_1(x, dy) \\
&= \int \{f(y) - f(x)\}^2 \min\left\{1, \frac{\pi(y)q_1(y,x)}{\pi(x)q_1(x,y)}\right\} \pi(x)q_1(x,y)dx\,dy \\
&= \int \{f(y) - f(x)\}^2 \min\left\{\pi(x)q_1(x,y), \pi(y)q_1(y,x)\right\} dx\,dy \\
&\geq \gamma \int \{f(y) - f(x)\}^2 \min\left\{\pi(x)q_2(x,y), \pi(y)q_2(y,x)\right\} \\
&= \gamma \int \{f(y) - f(x)\}^2 \min\left\{1, \frac{\pi(y)q_2(y,x)}{\pi(x)q_2(x,y)}\right\} \pi(x)q_2(x,y)dx\,dy \\
&= \gamma \int \{f(y) - f(x)\}^2 \pi(dx) P_2(x, dy).
\end{aligned}$$

The result follows from the Dirichlet forms characterization of spectral gaps in (3). □

To find upper bounds we use the notion of *conductance* for a Markov chain. Define the conductance of a set $K \in \mathcal{B}$ with $0 < \pi(K) \leq 1/2$ for a π-reversible Markov chain with transition kernel P as

$$\Phi(K) := \frac{\int_K \pi(dx) P(x, K^c)}{\pi(K)},$$

which is the conditional probability $\mathbb{P}(X^{(t+1)} \in K^c | X^{(t)} \in K)$ provided $X^{(t)} \sim \pi(\cdot)$. Recall the spectral gap bound for P that for any such K

$$\mathrm{Gap}(P) \leq 2\Phi(K). \tag{1.2}$$

This can be seen directly by setting $g(x) = \pi(K^C)\mathbb{I}(x \in K) - \pi(K)\mathbb{I}(x \in K^c)$, letting $f(x) := g(x)/\int g(x)^2 \pi(dx)$ and computing the Dirichlet form of f using (3). Here $\mathbb{I}(\cdot)$ denotes the indicator function.

2. Change of variables and isomorphic Markov chains

In this section we provide two lemmas showing that bijective mappings do not change the spectral gaps of Markov chains, nor the Metropolis-Hastings dynamics. These lemmas will allow us to prove the results of Section 2 working with the equivalent formulation where the target is fixed and the proposal distribution is changing, rather than having a target that changes with λ. This will in turn allow us to exploit results such as Lemma 1.1, thus significantly simplifying the proofs.

We follow the terminology of Johnson and Geyer (2013), and introduce the notion of *isomorphic* Markov chains. Intuitively, two Markov chains $(X^{(t)})_{t \geq 1}$ and $(Y^{(t)})_{t \geq 1}$ are isomorphic if $(\phi(X^{(t)}))_{t \geq 1}$ is equal in distribution to $(Y^{(t)})_{t \geq 1}$ for some bijective map ϕ. More formally, let $(X^{(t)})_{t \geq 1}$ and $(Y^{(t)})_{t \geq 1}$ be Markov chains with transition kernels P and K and state spaces $(S, \mathcal{A})$ and $(T, \mathcal{B})$, respectively. We say that $(X^{(t)})_{t \geq 1}$ and $(Y^{(t)})_{t \geq 1}$ are isomorphic if there exists a bijective function ϕ from S to T such that

$$P(x, A) = K(\phi(x), \phi(A)) \qquad x \in S,\ A \in \mathcal{A}. \tag{2.1}$$

Equation (2.1) means that $K(\phi(x), \cdot)$ is the push-forward of $P(x, \cdot)$ under ϕ for every $x \in \mathbb{R}^d$, which we write as $K = \phi \circ P$. We will use $\circ$ to denote the push-forward operator for both probability distributions and transition kernels, so that $(\phi \circ \pi)(B) = \pi(\phi^{-1}(B))$ and $(\phi \circ P)(y, B) = P(\phi^{-1}(y), \phi^{-1}(B))$.

Isomorphic Markov chains share the same convergence behaviour and, in particular, they have the same L^2-spectral gap, as stated in the following lemma (see Lemma 1 of Papaspilioupulus et al. (2019) for a proof of analogous results).

Lemma 2.1. *Isomorphic Markov chains have the same L^2-spectral gap.*

In the following we will exploit the fact that the Metropolis-Hastings (MH) algorithm preserves isomorphisms of Markov chains under transformations of both the target and candidate distributions, as shown by the following lemma.

Lemma 2.2. *Let $\phi : S \to T$ be a bijective function and $(X^{(t)})_{t \geq 1}$ and $(Y^{(t)})_{t \geq 1}$ be Metropolis-Hastings Markov chains defined on $(S, \mathcal{A})$ and $(T, \mathcal{B})$ with target distributions*

π and $\phi \circ \pi$, respectively, and proposal kernels Q and $\phi \circ Q$, respectively. Then $(X^{(t)})_{t\geq 1}$ and $(Y^{(t)})_{t\geq 1}$ are isomorphic Markov chains.

Proof. Let $\mu^\phi(dy, dy') := (\phi \circ \pi)(dy')(\phi \circ Q)(y', dy)$ and $\mu_T^\phi(dy, dy') := \mu^\phi(dy', dy)$. Then using Proposition 1 of Tierney (1998) there exists a set $R^\phi \in \mathcal{B}\times\mathcal{B}$ such that μ^ϕ and μ_T^ϕ are mutually absolutely continuous on R^ϕ and mutually singular on its complement. The Radon-Nikodym derivative $d\mu^\phi/d\mu_T^\phi(y, y')^T$ is therefore finite and positive when restricted to R^ϕ. Let $r^\phi(y, y') := d\mu^\phi/d\mu_T^\phi(y, y')$ if $(y, y') \in R^\phi$ and $r^\phi(y, y') := 0$ otherwise. Then the Metropolis–Hastings acceptance probability for the chain $(Y_t)_{t\geq 1}$ can be written $\alpha^\phi(y, y') := \min(1, r^\phi(y, y'))$. Similarly, letting $\mu(dx, dx') := \pi(dx')Q(x', dx)$ and $\mu_T(dx, dx') := \mu(dx', dx)$ the acceptance probability for $(X_t)_{t\geq 1}$ can be written $\alpha(x, x') := \min(1, r(x, x'))$ where $r(x, x') := d\mu/d\mu_T(x, x')$ when $(x, x') \in R \in S \times S$ and 0 otherwise, with R defined analogously to R^ϕ for the measures μ and μ_T.

Note first that from the definitions of push-forward measure and transition kernel given above that $\mu^\phi(A, B) = \mu(\phi^{-1}(A), \phi^{-1}(B))$ and $\mu_T^\phi(A, B) = \mu_T(\phi^{-1}(A), \phi^{-1}(B))$ for any $(A, B) \in \mathcal{B} \times \mathcal{B}$. From this it follows that $R \in \mathcal{A} \times \mathcal{A}$ is the pre-image under ϕ of $R^\phi \in \mathcal{B} \times \mathcal{B}$, and further that

$$\alpha^\phi(y, y') = \min(1, r^\phi(y, y')) = \min(1, r(\phi^{-1}(y), \phi^{-1}(y')) = \alpha(\phi^{-1}(y), \phi^{-1}(y')).$$

Denoting the transition kernels of $(X^{(t)})_{t\geq 1}$ and $(Y^{(t)})_{t\geq 1}$ as P and K respectively, it therefore holds that

$$\begin{aligned} K(y, B) =&\delta_B(y) \int_T (1 - \alpha^\phi(y, y'))\phi \circ Q(y, dy') + \int_B \alpha^\phi(y, y')\phi \circ Q(y, dy') \\ =&\delta_{\phi^{-1}(B)}(\phi^{-1}(y)) \int_S (1 - \alpha(\phi^{-1}(y), x'))Q(\phi^{-1}(y), dx') \\ &+ \int_{\phi^{-1}(B)} \alpha^\phi(\phi^{-1}(y), x')Q(\phi^{-1}(y), dx') \\ =&P(\phi^{-1}(y), \phi^{-1}(B)) \end{aligned}$$

meaning that $(X^{(t)})_{t\geq 1}$ and $(Y^{(t)})_{t\geq 1}$ are isomorphic. □

3. Proofs

Throughout the proofs we often use $\|\cdot\|$ to denote the standard euclidean norm $\|\cdot\|_2$.

3.1. Proofs for Section 2

Proof (Proof of Proposition 1). We first establish that $\mu(\delta_\lambda) \geq \mu(\delta)$ whenever $\lambda \leq \lambda_0$ for some $\lambda_0 > 0$. In cases (i) and (iii) $\mu(z)$ is monotonically decreasing in $\|z\|_2^2$. So when $\lambda < 1$ it holds that

$$\|\delta_\lambda\|_2^2 = \sum_{i=1}^{d} (y_i - x_i)^2 + \lambda^2(y_1 - x_1)^2 \leq \|\delta\|_2^2,$$

which proves the condition for $\lambda_0 = 1$. In case (ii) $\mu(z)$ is monotonically decreasing in $\|z\|_1$, so again $\|\delta_\lambda\|_1 = \lambda|y_1 - x_1| + \sum_{i=2}^d |y_i - x_i| \leq \|\delta\|$ when $\lambda < 1$. The statement that $\sup_{z_1 \in \mathbb{R}} \mu_1(z_1) < \infty$ follows by noting that in all three cases the marginal μ_1 is known in closed form and is, respectively, a Gaussian, Laplace and Student's t distribution, all of which have bounded density. □

Proof (Proof of Theorem 1). Instead of studying directly P_λ^R, we will study the transition kernel $\tilde{P}_\lambda^R$ corresponding to a Metropolis-Hastings (MH) algorithm with proposal $\phi \circ Q^R$ and target $\phi \circ \pi^{(\lambda)}$, for some bijective $\phi : \mathbb{R}^d \to \mathbb{R}^d$. By Lemma 2.2, $\tilde{P}_\lambda^R$ and P_λ^R induce isomorphic Markov chains and thus by Lemma 2.1 we have $\text{Gap}(\tilde{P}_\lambda^R) = \text{Gap}(P_\lambda^R)$. We consider ϕ given by $\phi(x_1, \dots, x_d) = (\lambda^{-1}x_1, x_2, \dots, x_d)$. It follows that $\phi \circ \pi^{(\lambda)} = \pi$ and that $\tilde{Q}_\lambda^R = \phi \circ Q$ satisfies $\tilde{Q}_\lambda^R(x, dy) = \tilde{q}_\lambda^R(x, y)dy$ with

$$\tilde{q}_\lambda^R(x, y) := \frac{\lambda}{\sigma^d}\mu\left(\frac{\delta_\lambda}{\sigma}\right), \tag{3.1}$$

and δ_λ defined as in equation (5) of the paper.

First we show that for all $\lambda \leq \lambda_0$ and all $x, y \in \mathbb{R}^d$ it holds that $\tilde{q}_\lambda^R(x, y) \geq \lambda \tilde{q}_1^R(x, y)$, where $\lambda_0 > 0$ is the value defined in Condition 1. From (3.1), we have

$$\frac{\tilde{q}_\lambda^R(x, y)}{\tilde{q}_1^R(x, y)} = \lambda \frac{\mu(\delta_\lambda/\sigma)}{\mu(\delta/\sigma)}. \tag{3.2}$$

Condition 1 guarantees $\mu(\delta_\lambda/\sigma) \geq \mu(\delta/\sigma)$ for all $\lambda \leq \lambda_0$, which together with (3.2) gives $\tilde{q}_\lambda^R(x, y) \geq \lambda \tilde{q}_1^R(x, y)$. Combining the latter inequality with Lemma 1.1 gives

$$\text{Gap}(\tilde{P}_\lambda^R) \geq \lambda \text{Gap}(\tilde{P}_1^R) = \Theta(\lambda) \qquad \text{as } \lambda \downarrow 0\,.$$

To show that $\text{Gap}(\tilde{P}_\lambda^R) \leq \Theta(\lambda)$, take $X^{(t)} \sim \pi(\cdot)$ and $X^{(t+1)}|X^{(t)} \sim \tilde{P}_\lambda^R(X^{(t)}, \cdot)$. We consider the set $K := \{y \in \mathbb{R}^d : |y_1| > k\}$, with k chosen such that $0 < \pi(K) < 1/2$ (since $\pi(\cdot)$ is defined on a Polish space, it is tight, meaning this is always possible). Recall from (1.2) that $\text{Gap}(\tilde{P}_\lambda^R) \leq 2\mathbb{P}(X^{(t+1)} \in K^c \,|\, X^{(t)} \in K)$. We have

$$\begin{aligned}
\mathbb{P}(X^{(t+1)} \in K^c \,\big|\, X^{(t)} \in K) \leq& \mathbb{P}(|X_1^{(t)} + \sigma\lambda^{-1}\xi_1| \leq k \,\big|\, X^{(t)} \in K) \\
=& \mathbb{P}(-X_1^{(t)} - k \leq \sigma\lambda^{-1}\xi_1 \leq -X_1^{(t)} + k \,\big|\, X^{(t)} \in K) \\
\leq& \sigma^{-1}\lambda 2k \sup_{z_1 \in \mathbb{R}} \mu_1(z_1)\,,
\end{aligned}$$

where ξ_1 is the first component of $\xi \sim \mu$. Conditon 1 implies $\sup_{z_1 \in \mathbb{R}} \mu_1(z_1) < \infty$, giving that $\text{Gap}(\tilde{P}_\lambda^R) \leq \Theta(\lambda)$ for $\lambda \downarrow 0$, as desired. □

Proof (Proof of Theorem 2). This follows directly from the proof of Theorem 4 below, by noting that setting $L = 1$ in Hamiltonian Monte Carlo gives the Langevin algorithm. □

Proof (Proof of Theorem 3). Similarly to the proof of Theorem 1, instead of studying directly P_λ^M, we will study the MH transition kernel $\tilde{P}_\lambda^M$ with proposal $\phi \circ Q_\lambda^M$

and target $\phi \circ \pi^{(\lambda)}$. Lemma 2.2 and Lemma 2.1 imply $\text{Gap}(\tilde{P}_\lambda^M) = \text{Gap}(P_\lambda^M)$. We consider the same ϕ as in the proof of Theorem 1, which we write as $\phi(x) = \Sigma_\lambda^{1/2} x$ with

$$\Sigma_\lambda = \begin{pmatrix} \lambda^{-2} & (0,\dots,0) \\ (0,\dots,0)^T & \mathbb{I}_{d-1} \end{pmatrix}.$$

We have $\pi = \phi \circ \pi^{(\lambda)}$ as stated above. Also, $\tilde{Q}_\lambda^M = \phi \circ Q_\lambda^M$ satisfies $\tilde{Q}_\lambda^M(y,\cdot) = N(y + \frac{\sigma^2}{2}\Sigma_\lambda \nabla \log \pi(y), \sigma^2 \Sigma_\lambda)$ where Σ_λ is as above. This is a fairly standard calculation, analogous to the derivation of the preconditioned MALA algorithm with preconditioning matrix $\Sigma_\lambda^{1/2}$, which we report here for completeness. By definition of ϕ and $Q_\lambda^M(x,\cdot) = N(x + \frac{\sigma^2}{2}\nabla \log \pi^{(\lambda)}(x), \sigma^2 \mathbb{I}_d)$, we have $\phi \circ Q_\lambda^M(x,\cdot) = N\left(\phi\big(x + \frac{\sigma^2}{2}\nabla \log \pi^{(\lambda)}(x)\big), \sigma^2\Sigma_\lambda\right)$ for each $x \in \mathbb{R}^d$. Also, since $\log \pi^{(\lambda)}(x) = \log \pi(\phi(x)) + const$ and $\phi(x) = \Sigma_\lambda^{1/2} x$, we have $\nabla \log \pi^{(\lambda)}(x) = \Sigma_\lambda^{1/2} \nabla \log \pi(\phi(x))$. Therefore

$$\phi\left(x + \frac{\sigma^2}{2}\nabla \log \pi^{(\lambda)}(x)\right) = \Sigma_\lambda^{1/2}\left(x + \frac{\sigma^2}{2}\Sigma_\lambda^{1/2}\nabla \log \pi(\phi(x))\right) = \phi(x) + \frac{\sigma^2}{2}\Sigma_\lambda \nabla \log \pi(\phi(x))$$

meaning that $\tilde{Q}_\lambda^M(\phi(x),\cdot)$ is the push-forward of $Q_\lambda^M(x,\cdot)$ under ϕ for every $x \in \mathbb{R}^d$, as desired.

We now prove $\text{Gap}(\tilde{P}_\lambda^M) \leq \Theta(e^{-\lambda^{-\alpha}})$ as $\lambda \downarrow 0$ for some $\alpha > 0$. We take $\sigma = 1$ for simplicity of notation (or otherwise replace λ by $\sigma^{-1}\lambda$) and we assume $\lambda < 1$ without loss of generality (we are studying a limit $\lambda \downarrow 0$). Let $(X^{(t)})_{t=1}^\infty$ be a Markov chain with transition kernel $\tilde{P}_\lambda^M$ started in stationarity. We consider the sets $A_\lambda := \{y \in \mathbb{R}^d : |y_1| \leq \lambda^{-1/(2\tilde{\gamma})}\}$, where $\tilde{\gamma} = \max\{1,\gamma\}$, and $K := \{y \in \mathbb{R}^d : |y_1| > k\}$, with k chosen such that $0 < \pi(K) < 1/2$. Given

$$\epsilon \in \left(0, \liminf_{|x_1|\to\infty}\left(\inf_{(x_2,\dots,x_d)\in\mathbb{R}^{d-1}}\left|\frac{\partial \log \pi(x)}{\partial x_1}\right| \|x\|^\gamma\right)\right),$$

Condition 3(i) implies that we can choose k large enough such that

$$\left|\frac{\partial \log \pi(x)}{\partial x_1}\right| \|x\|^\gamma \geq \epsilon \qquad \text{for all } x \in K.$$

We will now show $\mathbb{P}(X^{(t+1)} \in K^c \mid X^{(t)} \in K) \leq \Theta(e^{-\lambda^{-\alpha}})$ for some $\alpha > 0$ as $\lambda \downarrow 0$. Note that

$$\begin{aligned}\pi(K)\mathbb{P}(X^{(t+1)} \in K^c | X^{(t)} \in K) = &\ \mathbb{P}(X^{(t+1)} \in K^c | X^{(t)} \in K \cap A_\lambda)\mathbb{P}(X^{(t)} \in K \cap A_\lambda) \\ &+ \mathbb{P}(X^{(t+1)} \in K^c | X^{(t)} \in K \cap A_\lambda^c)\mathbb{P}(X^{(t)} \in K \cap A_\lambda^c),\end{aligned}$$

meaning that

$$\pi(K)\mathbb{P}(X^{(t+1)} \in K^c | X^{(t)} \in K) \leq \mathbb{P}(X^{(t+1)} \in K^c | X^{(t)} \in K \cap A_\lambda) + \mathbb{P}(X^{(t)} \in K \cap A_\lambda^c).$$

Condition 3(ii) implies $\mathbb{P}(X^{(t)} \in K \cap A_\lambda^c) \leq \mathbb{P}(X^{(t)} \in A_\lambda^c) \leq \Theta(e^{-\lambda^{-\beta/(2\tilde{\gamma})}})$.

Also, given $Y = (Y_1, \dots, Y_d)$ with $Y|X^{(t)} \sim \tilde{Q}_h^M(X^{(t)}, \cdot)$, we have

$$\mathbb{P}(X^{(t+1)} \in K^c \,|\, X^{(t)} \in K \cap A_\lambda) \leq \mathbb{P}(|Y_1| \leq k \,|\, X^{(t)} \in K \cap A_\lambda)\,.$$

Denote $\frac{\partial}{\partial x_1} \log \pi(x)$ by $\partial_1(x)$ for brevity. If $X^{(t)} \in K \cap A_\lambda$ we have $|X_1^{(t)}| \leq \lambda^{-1/(2\tilde{\gamma})} \leq \lambda^{-1/2}$ and

$$|\partial_1(X^{(t)})| \geq \epsilon \|X^{(t)}\|^{-\gamma} \geq \epsilon \lambda^{\gamma/(2\tilde{\gamma})} \geq \epsilon \lambda^{1/2}\,,$$

which imply

$$\begin{aligned}|Y_1| &= |X_1^{(t)} + \lambda^{-2}\partial_1(X^{(t)}) + \lambda^{-1}\xi_1| \\ &\geq \lambda^{-2}|\partial_1(X^{(t)})| - \lambda^{-1}|\xi_1| - |X_1^{(t)}| \\ &\geq \lambda^{-3/2}\epsilon - \lambda^{-1}|\xi_1| - \lambda^{-1/2}\,,\end{aligned}$$

where $\xi_1 \sim N(0,1)$. It follows that

$$\begin{aligned}\mathbb{P}(|Y_1| \leq k \,|\, X^{(t)} \in K \cap A_\lambda) \leq& \mathbb{P}(\lambda^{-3/2}\epsilon - \lambda^{-1}|\xi_1| - \lambda^{-1/2} \leq k \,|\, X^{(t)} \in K \cap A_\lambda) \\ =& \mathbb{P}(|\xi_1| \geq \epsilon\lambda^{-1/2} - \lambda^{1/2} - k\lambda)\,.\end{aligned}$$

Since $\mathbb{P}(|\xi_1| \geq t) \leq \exp(-t^2/2)$ for every $t > 0$ (which follows from standard bounds on Gaussian tails and $\xi_1 \sim N(0,1)$) and $\epsilon\lambda^{-1/2} - \lambda^{1/2} - k\lambda \geq 2\lambda^{-1/3}$ eventually as $\lambda \downarrow 0$, it follows

$$\mathbb{P}(|Y_1| \leq k \,|\, X^{(t)} \in K \cap A_\lambda) \leq \Theta(e^{-\lambda^{-2/3}}) \qquad \text{as } \lambda \downarrow 0\,.$$

Combining the inequalities above and noting that $\pi(K)$ does not depend on λ, it follows that $\mathbb{P}(X^{(t+1)} \in K^c | X^{(t)} \in K) \leq \Theta(e^{-\lambda^{-\alpha}})$ for $\alpha = \min\{\beta/(2\tilde{\gamma}), 2/3\} > 0$ as $\lambda \downarrow 0$. Finally, the conductance bound in (1.2) imply $\text{Gap}(\tilde{P}_\lambda^M) \leq \Theta(e^{-\lambda^{-\alpha}})$ as $\lambda \downarrow 0$. □

Proof (Proof of Theorem 4). Similarly to the case of the random walk and Langevin schemes, we will study the MH transition kernel $\tilde{P}_\lambda^H$ with proposal $\phi \circ Q_\lambda^H$ and target $\phi \circ \pi^{(\lambda)}$, and exploit the fact that $\text{Gap}(\tilde{P}_\lambda^H) = \text{Gap}(P_\lambda^H)$ by Lemma 2.2 and Lemma 2.1. Considering $\phi(x) = \Sigma_\lambda^{1/2} x$ as above we have $\pi = \phi \circ \pi^{(\lambda)}$ and $\tilde{Q}_\lambda^H = \phi \circ Q_\lambda^H$ evolving according to a preconditioned HMC algorithm as follows. Writing the current point $x \in \mathbb{R}^d$ as $x(0)$, as in Section 2.3.3 of the the paper, the proposal $y := x(L) \sim \tilde{Q}_\lambda^H(x, \cdot)$ is obtained using the update

$$x(L) = x(0) + \sigma^2 \left(\frac{L}{2}\Sigma_\lambda \nabla \log \pi(x(0)) + \sum_{j=1}^{L-1} (L-j)\Sigma_\lambda \nabla \log \pi\,(x(j)) \right) + L\sigma\Sigma_\lambda^{1/2}\xi(0), \tag{3.3}$$

where each $x(j)$ is defined recursively in the same manner, and $\xi(0) \sim N(0, \mathbb{I}_d)$. It is easy to check that $\tilde{Q}_\lambda^H = \phi \circ Q_\lambda^H$ using the same calculations as in the proof of Theorem 3.

We now prove $\text{Gap}(\tilde{P}_\lambda^H) \leq \Theta(e^{-\lambda^{-\alpha}})$ as $\lambda \downarrow 0$ for some $\alpha > 0$. To simplify the notation in the following we prove the equivalent statement that $\text{Gap}(\tilde{P}_{h^{-1}}^H) \leq \Theta(e^{-h^\alpha})$

as $h \to \infty$. Fix $\delta \in (0, (1-q)/2)$ with q defined in Condition 2 and consider the sets $A_h := \{y \in \mathbb{R}^d : |y_1| < k + h\}$ and $K := \{y \in \mathbb{R}^d : |y_1| > k\}$. Here k is chosen large enough that Lemma 3.1 below is satisfied and that $0 < \pi(K) < 1/2$, which can always be done thanks to the tightness and positiveness of π (see above). Lemma 3.1 implies that if $X^{(t)} \in K \cap A_h$, $|\xi_1| \leq h^{1-\delta}$ and $h \geq h_0$, where $h_0 = \lambda_0^{-1}$ with λ_0 defined as in Lemma 3.1, then $X^{(t+1)} \in K$. We now upper bound the probability $\mathbb{P}(X^{(t+1)} \in K^c | X^{(t)} \in K)$. First note that

$$\begin{aligned}\mathbb{P}(X^{(t+1)} \in K^c, X^{(t)} \in K) = \mathbb{P}(X^{(t+1)} \in K^c | X^{(t)} \in K \cap A_h)\mathbb{P}(X^{(t)} \in K \cap A_h)\\ +\mathbb{P}(X^{(t+1)} \in K^c | X^{(t)} \in K \cap A_h^c)\mathbb{P}(X^{(t)} \in K \cap A_h^c),\end{aligned}$$

which implies

$$\mathbb{P}(X^{(t+1)} \in K^c, X^{(t)} \in K) \leq \mathbb{P}(X^{(t+1)} \in K^c | X^{(t)} \in K \cap A_h) + \mathbb{P}(X^{(t)} \in K \cap A_h^c).$$

Breaking out the first term on the right-hand side gives

$$\begin{aligned}&\mathbb{P}(X^{(t+1)} \in K^c | X^{(t)} \in K \cap A_h) \leq \\ &\mathbb{P}(X^{(t+1)} \in K^c | X^{(t)} \in K \cap A_h, |\xi_1| \leq h^{1-\delta})\mathbb{P}(|\xi_1| \leq h^{1-\delta}) + \mathbb{P}(|\xi_1| > h^{1-\delta}),\end{aligned}$$

which, using the result of Lemma 3.1, reduces to

$$\mathbb{P}(X^{(t+1)} \in K^c | X^{(t)} \in K \cap A_h) \leq \mathbb{P}(|\xi_1| > h^{1-\delta}) \,.$$

Hence we obtain the overall bound

$$\mathbb{P}(X^{(t+1)} \in K^c, X^{(t)} \in K) \leq \mathbb{P}(|\xi_1| > h^{1-\delta}) + \mathbb{P}(X^{(t)} \in K \cap A_h^c).$$

Using standard bounds on Gaussian tails and $\xi_1 \sim N(0,1)$, we have $\mathbb{P}(|\xi_1| > h^{1-\delta}) \leq \exp(-h^{2(1-\delta)}/2)$. Also, from Lemma 3.3, we have $\mathbb{P}(X^{(t)} \in K \cap A_h^c) \leq \Theta(e^{-\gamma h^{1+q} - q\log(h)})$ as $h \to \infty$ for some $\gamma \in (0, \infty)$. Hence, since $\delta < (1-q)/2$ and $\mathbb{P}(X^{(t)} \in K) = \pi(K)$ is constant with respect to h, we obtain

$$\mathbb{P}(X^{(t+1)} \in K^c | X^{(t)} \in K) \leq \Theta\left(e^{-\gamma h^{1+q} - q\log(h)}\right) \qquad \text{as } h \to \infty\,.$$

Finally, the conductance bound in (1.2) gives

$$\mathrm{Gap}(\tilde{P}^H_{h^{-1}}) \leq \Theta\left(e^{-\gamma h^{1+q} - q\log(h)}\right) \qquad \text{as } h \to \infty\,.$$

□

Proof (Proof of Proposition 2). For the Langevin case, standard results on the total variation distance between two Gaussian measures with differing means reveals that

$$\|Q_\lambda^M(x,\cdot) - Q^R(x,\cdot)\|_{TV} = 1 + \frac{1}{\sqrt{2\pi}}\int_0^t e^{-u^2/2}du.$$

where $t = \sigma|\nabla \log \pi(x/\lambda)|/(4\lambda)$. Because $\nabla \log \pi$ is bounded in a neighbourhood of zero, for large enough λ we can write $t \leq C/\lambda$ for some $C < \infty$. Then note that as $\lambda \uparrow \infty$

$$\int_0^{C/\lambda} e^{-u^2/2} du \leq \frac{C}{\lambda}.$$

For the Barker case, note that the total variation distance here can be written

$$\frac{1}{2} \int \mu_\sigma(z) |2g(e^{\nabla \log \pi(x/\lambda) z/\lambda}) - 1| dz,$$

where $g(t) = 1/(1 + t^{-1})$. Setting $u := \nabla \log \pi(x) z$, a Taylor series expansion about $u = 0$ of $2g(u)$ is

$$2g(u) = 1 + \frac{u}{2} + g''(\xi) u^2,$$

for some ξ satisfying $|\xi| \leq |\nabla \log \pi(x) z|$, using the Lagrange form of the remainder. Substituting this into the integral and simplifying gives

$$\begin{aligned}\frac{1}{2} \int \mu_\sigma(z) \left| \frac{u}{2} + g''(\xi) u^2 \right| dz \leq \frac{1}{4\lambda} |\nabla \log \pi(x/\lambda)| \int |z| \mu_\sigma(z) dz \\ + \frac{1}{2\lambda^2} \nabla \log \pi(x/\lambda)^2 \int |g''(\xi)| z^2 \mu_\sigma(z) dz.\end{aligned}$$

For large enough λ the boundedness assumption allows us to write $|\nabla \log \pi(x/\lambda)| \leq c$ for some $c < \infty$. In addition note that $g''(\xi) = 2e^{-2\xi}/(1+e^{-\xi})^3 - e^{-\xi}/(1+e^{-\xi})^2$, and so $\sup_{\xi \in \mathbb{R}} |g''(\xi)| = (6\sqrt{3})^{-1}$. Substituting into the bound and evaluating the two integrals gives an upper bound to the total variation distance of

$$\left(\frac{c}{4} \sqrt{\frac{2\sigma^2}{\pi}} \right) \lambda^{-1} + \left(\frac{c^2 \sigma^2}{12\sqrt{3}} \right) \lambda^{-2},$$

which is $\Theta(1/\lambda)$ as $\lambda \uparrow \infty$, as desired.

□

3.1.1. Lemmas used to prove Theorem 4

LEMMA 3.1. *Assume Condition 2 and let $\delta \in (0,1)$. For every $L \geq 1$ there exist $\lambda_0 > 0$ and a large enough k such that for every $\lambda \leq \lambda_0$, $|\xi_1| \leq \lambda^{-(1-\delta)}$ and $|x_1(0)| \in [k, k+\lambda^{-1})$ it holds that $|x_1(L)| \geq k$, where $x(L)$ is defined in* (3.3).

PROOF. Recall that, for each $i \geq 1$, $x_1(i)$ is implicitly a function of the starting location $x_1(0)$, the parameter λ and the noise ξ_1. For notational convenience, in the following we set $h = \lambda^{-1}$ and study the limit $h \uparrow \infty$. In order to prove the thesis it is sufficient to show that for fixed, sufficiently large $k > 0$ we have

$$\inf_{\xi_1, x_1(0)} |x_1(L)| \to \infty \qquad \text{as } h \uparrow \infty, \tag{3.4}$$

where ξ_1 and $x_1(0)$ in the infimum are restricted as in the lemma's statement, i.e. $\xi_1 \in (-h^{1-\delta}, h^{1-\delta})$ and $x_1(0) \in (-(k+h), -k] \cup [k, k+h)$. In order to prove (3.4) we will show that for all $i \geq 1$, as $h \uparrow \infty$ we have

$$\Theta(h^{2\sum_{j=0}^{i-1} q^j}) \leq \inf |x_1(i)| \leq \sup |x_1(i)| \leq \Theta(h^{q^i + 2\sum_{j=0}^{i-1} q^j}), \tag{3.5}$$

$$\Theta(h^{2\sum_{j=1}^{i} q^j}) \leq \inf |\partial_1(i)| \leq \sup |\partial_1(i)| \leq \Theta(h^{q^{i+1} + 2\sum_{j=1}^{i} q^j}), \tag{3.6}$$

where infima and suprema run over $\xi_1 \in (-h^{1-\delta}, h^{1-\delta})$ and $x_1(0) \in (-(k+h), -k] \cup [k, k+h)$ as in (3.4), and $\partial_1(i)$ stands for $\partial \log \pi_1 / \partial x_1(x_1(i))$. Note that, for any $i \geq 1$, (3.6) is implied by (3.5) thanks to $\inf |x_1(1)| \to \infty$ as $h \to \infty$ and (7). Thus it suffices to prove that (3.5) holds for all $i \geq 1$, which we will do by induction over i.

In the following, k is chosen large enough that $c|x_1|^q \leq |\partial \log \pi_1 / \partial x_1(x_1)| \leq C|x_1|^q$ for some $0 < c \leq C < \infty$ and all $|x_1| > k$, which can be done by (7). Also, unless otherwise stated, we assume $\xi_1 \in (-h^{1-\delta}, h^{1-\delta})$ and $x_1(0) \in (-(k+h), -k] \cup [k, k+h)$, and all infima and suprema are taken over those sets.

Considering $i = 1$, we have $x_1(1) = x_1(0) + h\xi_1 + (h^2/2)\partial_1(0)$, which implies

$$\frac{h^2}{2}|\partial_1(0)| - |x_1(0)| - h|\xi_1| \leq |x_1(1)| \leq \frac{h^2}{2}|\partial_1(0)| + |x_1(0)| + h|\xi_1|.$$

Then, since $|\xi_1| \in (0, h^{1-q})$, $|x_1(0)| \in [k, k+h)$ and $ck^q \leq c|x_1(0)|^q \leq |\partial_1(0)| \leq C|x_1(0)|^q \leq C(h+k)^q$, we have

$$\Theta(h^2) = \frac{h^2}{2}ck^q - (k+h) - h^{2-\delta} \leq |x_1(1)| \leq \frac{h^2}{2}C(h+k)^q + (k+h) + h^{2-q} = \Theta(h^{2+q})$$

meaning that (3.5) is satisfied for $i = 1$.

We then show that if (3.5) and (3.6) hold for $i = 1, \ldots, \ell - 1$, where $\ell \geq 2$, then they also hold for $i = \ell$. First note that when $\ell \geq 2$, (3.3) implies

$$x_1(\ell) = x_1(\ell - 1) + h\xi_1 + \frac{h^2}{2}\partial_1(0) + h^2 \sum_{j=1}^{\ell-1} \partial_1(j). \tag{3.7}$$

From (3.7) and $|\xi_1| \in (0, h^{1-q})$, we can deduce that

$$h^2|\partial_1(\ell-1)| - |x_1(\ell-1)| - h^2 \sum_{j=0}^{\ell-2} |\partial_1(j)| \leq |x_1(\ell)| \leq |x_1(\ell-1)| + h^{2-q} + h^2 \sum_{j=0}^{\ell-1} |\partial_1(j)|. \tag{3.8}$$

Combining the lower bound in (3.8) with (3.5) and (3.6) for $i = 1, \ldots, \ell - 1$ we obtain

$$\begin{aligned}\inf |x_1(\ell)| &\geq \inf h^2|\partial_1(\ell - 1)| - \sup \left(|x_1(\ell - 1)| + h^2 \sum_{j=0}^{\ell-2} |\partial_1(j)| \right) \\ &\geq \Theta(h^{2\sum_{j=0}^{\ell-1} q^j}) - \Theta(h^{q^{\ell-1} + 2\sum_{j=0}^{\ell-2} q^j}) = \Theta(h^{2\sum_{j=0}^{\ell-1} q^j}),\end{aligned}$$

where the last equality follows from $q^{\ell-1}+2\sum_{j=0}^{\ell-2} q^j \le 2\sum_{j=0}^{\ell-1} q^j$. Thus the lower bound in (3.5) holds also for $i=\ell$. Similarly, combining the upper bound in (3.8) with (3.5) and (3.6) for $i=1,\dots,\ell-1$ we obtain

$$\sup |x_1(\ell)| \le \sup\left(|x_1(\ell-1)| + h^{2-q} + h^2\sum_{j=0}^{\ell-1}|\partial_1(j)|\right)$$
$$\le \Theta(h^{q^{\ell-1}+2\sum_{j=0}^{\ell-2}q^j} + h^{2-q} + h^{q^i+2\sum_{j=0}^{\ell-1}q^j}) = \Theta(h^{q^i+2\sum_{j=0}^{\ell-1}q^j}),$$

where the last equality follows from $2-q \le q^{\ell-1}+2\sum_{j=0}^{\ell-2} q^j \le q^i + 2\sum_{j=0}^{\ell-1} q^j$. Thus the upper bound in (3.5) holds also for $i=\ell$ and the proof is complete. □

LEMMA 3.2. *Condition 2 (ii) implies that there exist t, c and C in $(0,\infty)$ such that*

$$\pi_1(x_1) \le Ce^{-c|x_1|^{1+q}}, \qquad \text{for all } |x_1| \ge t. \tag{3.9}$$

PROOF. Condition 2 implies that there exists $t, c \in (0,\infty)$ such that

$$\left|\frac{d}{dx_1}\log \pi_1(x_1)\right| \ge c|x_1|^{1+q}, \qquad \text{for all } |x_1| \ge t.$$

Since $\log \pi_1 \in C_1(\mathbb{R})$, the above implies that either

$$\frac{d}{dx_1}\log\pi_1(x_1) > cx_1^{1+q} \quad \text{or} \quad \frac{d}{dx_1}\log\pi_1(x_1) < -cx_1^{1+q},$$

holds for all $|x_1| \ge t$. Since $\int \pi_1(x_1)dx_1 = 1$ the latter option must be true. Computing the anti-derivative gives

$$\log\pi_1(x_1) \le -cx_1^{1+q} + \log C,$$

for some constant $\log C$. An analogous argument can be used in the case $x_1 \downarrow -\infty$, and the two combined give the result. □

LEMMA 3.3. *If Condition 2 holds and $X_1 \sim \pi_1(\cdot)$, then there exists $\gamma \in (0,\infty)$ such that*

$$\mathbb{P}(|X_1| > k+h) \le \Theta\left(e^{-\gamma h^{1+q} - q\log(h)}\right) \qquad \text{as } h \to \infty.$$

PROOF. Using Lemma 3.2, provided $k+h > t$ we have

$$\begin{aligned}\mathbb{P}(|X_1| > k+h) =& \int_{k+h}^{\infty}\pi_1(x_1)dx_1 + \int_{-\infty}^{-(k+h)}\pi_1(x_1)dx_1\\ \le& 2C\int_{k+h}^{\infty} e^{-cx_1^{1+q}}dx_1\\ =& 2C\frac{c^{-1/(q+1)}}{q+1}\Gamma\left(\frac{1}{1+q}, c(k+h)^{1+q}\right),\end{aligned}$$

where $\Gamma(a,b) := \int_b^\infty u^{a-1}e^{-u}du$ is the incomplete Gamma function. For the case $q > 0$ the upper bound of Gautschi (1959), which is described on pages 771-772 of Alzer (1997), states that for fixed $a \in (0,1)$ and $x > 0$ we have

$$\Gamma(a, x^{-a}) \leq e^{-x^{-a}} \frac{c_a}{a} \left((x^{-a} + c_a^{-1})^a - x \right),$$

where $c_a := \Gamma(1+a)^{1/(1-a)}$. Setting $C_2 := 2Cc^{-1/(q+1)}/(q+1)$, $a := 1/(1+q)$ and using this upper bound gives

$$\mathbb{P}(|X_1| > k+h) \leq e^{-c(k+h)^{1+q}} C_2 \frac{c_a}{a} \left[\left(c(k+h)^{\frac{1}{a}} + c_a^{-1} \right)^a - c^a(k+h) \right].$$

We use a Taylor series expansion of $f(c(k+h)^{1/a} + c_a^{-1})$ about $f(c(k+h)^{1/a})$, where $f(x) = x^a$. The terms each have a different power of h. This gives

$$(c(k+h)^{\frac{1}{a}} + c_a^{-1})^a = c^a(k+h) + c_a^{-1} a (c(k+h)^{\frac{1}{a}})^{a-1} + O(h^{(a-2)/a})$$

Since $a = 1/(1+q) < 1$ then $(a-1)/a = -q$ and $(a-2)/a = -(1+2q)$, and therefore

$$(c(k+h)^{\frac{1}{a}} + c_a^{-1})^a - c^a(k+h) = \Theta((c(k+h)^{\frac{1}{a}})^{a-1}) = \Theta(h^{-q}).$$

Combining with the above, we can write that for any fixed k and fixed $q > 0$, there exists $\gamma \in (0,\infty)$ such that as $h \uparrow \infty$

$$\mathbb{P}(|X_1| > k+h) \leq \Theta\left(e^{-\gamma h^{1+q} - q\log(h)}\right).$$

In the case $q = 0$ the integral $\int_{k+h}^\infty e^{-cx_1}dx_1 = e^{-c(k+h)}/c$ and the result is immediate. □

3.2. Proofs for Section 3

PROOF (PROOF OF PROPOSITION 3). Setting $y - x = z$, then $t(z) = e^{z\nabla \log \pi(x)}$ and $1/t(z) = e^{-z\nabla \log \pi(x)} = t(-z)$, meaning

$$\begin{aligned} Z(x) &= \int_{\mathbb{R}} \frac{t(z)}{1+t(z)} \mu_\sigma(z) dz \\ &= \int_0^\infty \left(\frac{t(z)}{1+t(z)} \mu_\sigma(z) + \frac{t(-z)}{1+t(-z)} \mu_\sigma(-z) \right) dz. \end{aligned}$$

Noting that $\mu_\sigma(z) = \mu_\sigma(-z)$ and $t(-z) = 1/t(z)$ then gives

$$Z(x) = \int_0^\infty \mu_\sigma(z) dz = \frac{1}{2}$$

which completes the proof. □

PROOF (PROOF OF PROPOSITION 4). Assume $y = x + b(x,z) \times z$ is generated using Algorithm 1. Then for any $A \in \mathcal{B}(\mathbb{R})$

$$\mathbb{P}[y \in A] = \mathbb{P}\left[\{z \in A - x\} \cap \{b(x,z) = 1\}\right] + \mathbb{P}\left[\{-z \in A - x\} \cap \{b(x,z) = -1\}\right].$$

Note that the second term on the right-hand side can be re-written

$$\mathbb{P}\left[\{z \in A - x\} \cap \{b(x, -z) = -1\}\right],$$

owing to the symmetry of μ_σ. Because of this, we can write

$$\begin{aligned}\mathbb{P}[y \in A] &= \int_{A-x} \frac{e^{z\nabla \log \pi(x)}}{1 + e^{z\nabla \log \pi(x)}} \mu_\sigma(z) dz + \int_{A-x} \frac{1}{1 + e^{-z\nabla \log \pi(x)}} \mu_\sigma(z) dz \\ &= 2\int_{A-x} \frac{e^{z\nabla \log \pi(x)}}{1 + e^{z\nabla \log \pi(x)}} \mu_\sigma(z) dz \\ &= Q^B(x, A)\end{aligned}$$

which completes the proof. □

Proof (Proof of Proposition 5). We establish a point-wise bound on the candidate transition densities of the two algorithms. Combining this with Lemma 1.1 gives an equivalent bound on the spectral gaps. To reach this point-wise bound, first note that the candidate transition density associated with the Random Walk algorithm is $q^R(x, x + z) = \mu_\sigma(z)$ for any $x, z \in \mathbb{R}^d$. Now, for the modified Barker proposal, the candidate density can be written

$$\begin{aligned}\check{q}^B(x, x + z) &= \mu_\sigma(z)\check{p}(x, z) + \mu_\sigma(-z)(1 - \check{p}(x, -z)) \\ &= \mu_\sigma(z)\left(\check{p}(x, z) - \check{p}(x, -z) + 1\right) \\ &= 2\check{p}(x, z)\mu_\sigma(z),\end{aligned}$$

where on the last line we have used that $\check{p}(x, -z) = 1 - \check{p}(x, z)$. Noting that $\tilde{p}(x, z) \leq 1$ establishes that $q^R(x, x + z) \geq \check{q}^B(x, x + z)/2$ for any $x, z \in \mathbb{R}^d$, and upon combining this with Lemma 1.1 the result follows. □

3.3. Proofs for Section 4

Interestingly, the proof of the lower bound of Theorem 5 is analogous to the one of Theorem 1, providing further insight into the similarity between the Barker scheme and random walk in terms of robustness to scales.

Proof (Proof of Theorem 5). As in the proof of Theorem 1, we write Q_λ^B to denote the Barker candidate kernel targeting $\pi^{(\lambda)}$, and $\tilde{Q}_\lambda^B(x, dy) := \tilde{q}_\lambda^B(x, y)dy$ to denote the isomorphic kernel defined as $\tilde{Q}_\lambda^B = \phi \circ Q_\lambda^B$, where ϕ is the same function used in the proof of Theorem 1. Also, we denote by P_λ^B and $\tilde{P}_\lambda^B$ the Metropolis-Hastings kernels with candidate kernels Q_λ^B and $\tilde{Q}_\lambda^B$, respectively, and target distributions $\pi^{(\lambda)}$ and π, respectively.

From (16) and (17) it follows that

$$\tilde{q}_\lambda^B(x, y) = 2^d \frac{\lambda}{\sigma^d} \mu\left(\frac{\delta_\lambda}{\sigma}\right) \prod_{i=1}^d \left(1 + e^{-\partial_i \log \pi(x)(y_i - x_i)}\right)^{-1}. \tag{3.10}$$

Here we are using μ to denote the d-dimensional distribution obtained by proposing each coordinate independently as in Section 3.3 of the paper. We therefore have

$$\frac{\tilde{q}_\lambda^B(x,y)}{\tilde{q}_1^B(x,y)} = \lambda\frac{\mu(\delta_\lambda/\sigma)}{\mu(\delta/\sigma)}\,, \tag{3.11}$$

which holds after noting that $(1+e^{-\partial_i \log\pi(x)(y_i-x_i)})$ does not depend on λ, and hence cancels in the ratio. Note that the expression above coincides with the expression for the random walk proposals in (3.2). Thus, arguing as in the proof of Theorem 1, we have that $\tilde{q}_\lambda^B(x,y) \geq \lambda\tilde{q}^B(x,y)$ for all $\lambda \leq \lambda_0$ and all $x, y \in \mathbb{R}^d$, where $\lambda_0 \leq 1$ is the value defined in Condition 1. Combining the latter inequality with Lemma 1.1 and using the isomorphism property between $\tilde{P}_\lambda^B$ and P_λ^B given in Lemmas 2.1 and 2.2, we obtain

$$\mathrm{Gap}(P_\lambda^B) \geq \lambda\mathrm{Gap}(P^B) = \Theta(\lambda) \qquad \text{as } \lambda \downarrow 0\,.$$

To show that $\mathrm{Gap}(P_\lambda^B) \leq \Theta(\lambda)$, note that $\tilde{q}_\lambda^B(x,y) \leq 2^d\tilde{q}_\lambda^R(x,y)$ for all $x, y \in \mathbb{R}^d$ by (3.10) and (4). Thus, Lemma 1.1 and Theorem 1 give $\mathrm{Gap}(P_\lambda^B) \leq 2^d\mathrm{Gap}(P_\lambda^R) = \Theta(\lambda)$ as $\lambda \downarrow 0$. □

3.3.1. Proof of Theorem 6

The following lemma, which is an extension of Theorem 4.1 of (Roberts and Tweedie, 1996), provides generic sufficient conditions for the geometric ergodicity of Metropolis–Hastings algorithms.

LEMMA 3.4. *Let P be a ϕ-irreducible and aperiodic Metropolis–Hastings kernel on $\mathbb{R}^d$ with proposal Q such that compact sets are small under P. If there exist a function $V : \mathbb{R}^d \to (0,\infty)$ such that $\sup_{x\in\mathbb{R}^d} \frac{QV(x)}{V(x)} < \infty$ and*

$$\liminf_{\|x\|\to+\infty} \int_{R^d} q(x,y)\alpha(x,y)\,dy > \limsup_{\|x\|\to\infty} \frac{QV(x)}{V(x)}\,, \tag{3.12}$$

then P is π-a.e. geometrically ergodic.

PROOF. We show that (3.12) implies the following Foster-Lyapunov drift conditions:

$$\sup_{x\in\mathbb{R}^d} \frac{PV(x)}{V(x)} < \infty \text{ and } \limsup_{\|x\|\to\infty} \frac{PV(x)}{V(x)} < 1\,,$$

which imply π-a.e. geometric ergodicity (see e.g. Theorem 3.1 and Lemma 3.5 of Jarner and Hansen (2000)). First note that

$$\begin{aligned}\frac{PV(x)}{V(x)} &= \int_{\mathbb{R}^d} \left(\frac{V(y)}{V(x)}\alpha(x,y) + 1 - \alpha(x,y)\right) q(x,y)dy \\ &\leq \int_{\mathbb{R}^d} \frac{V(y)}{V(x)} q(x,y)dy + \int_{\mathbb{R}^d} (1-\alpha(x,y))\, q(x,y)dy \leq \frac{QV(x)}{V(x)} + 1\,,\end{aligned}$$

which implies $\sup_{x\in\mathbb{R}^d} \frac{PV(x)}{V(x)} \leq \sup_{x\in\mathbb{R}^d} \frac{QV(x)}{V(x)} + 1 < \infty$. Also, the inequalities above imply

$$\frac{PV(x)}{V(x)} \leq 1 - \left(\int_{\mathbb{R}^d} \alpha(x,y)q(x,y)dy - \frac{QV(x)}{V(x)} \right) . \tag{3.13}$$

From (3.12) we have

$$\begin{aligned} 0 &< \liminf_{\|x\|\to+\infty} \int_{\mathbb{R}^d} q(x,y)\alpha(x,y)\,dy - \limsup_{\|x\|\to\infty} \frac{QV(x)}{V(x)} \\ &\leq \liminf_{\|x\|\to+\infty} \left(\int_{\mathbb{R}^d} q(x,y)\alpha(x,y)\,dy - \frac{QV(x)}{V(x)} \right) . \end{aligned} \tag{3.14}$$

Combining (3.13) and (3.14) we obtain $\limsup_{\|x\|\to\infty} \frac{PV(x)}{V(x)} < 1$, as desired. □

We will show that the conditions of Lemma 3.4 are satisfied when considering a Lyapunov function $V_s(x) = \exp(s\|x\|_\infty)$ based on the sup norm, $\|x\|_\infty = \sup_i |x_i|$.

In the following results we denote $\sup_{t>0} g(t)$ by M. We denote the log-target and its derivatives as $U(x) = \log \pi(x)$ and $U_i(x) = \frac{\partial}{\partial x_i} U(x)$, respectively. Condition 4 implies that $\nabla U(x) = f'(\|x\|)\frac{x}{\|x\|}$ and $U_i(x) = f'(\|x\|)\frac{x_i}{\|x\|}$ for $\|x\| > R$. Also, we denote the kernel $Q^{(g)}$ in (21) as Q for brevity and its density function as

$$q(x,y) = \prod_{i=1}^{d} \frac{g(e^{w_i U_i(x)})\mu_\sigma(w)}{Z_i(x)} = \prod_{i=1}^{d} q_i(w_i;x)\,, \tag{3.15}$$

where $w_i = y_i - x_i$ and $q_i(w_i;x) = g(e^{w_i U_i(x)})\mu_\sigma(w)/Z_i(x)$.

First, we provide some simple results on the behaviour of g, Z_i and q_i that will be useful afterwards.

Lemma 3.5. *Let $g : (0,\infty) \to (0,\infty)$ be bounded, non-decreasing and such that $g(t) = tg(1/t)$ for all $t > 0$. Then $g(t) \geq g(1)\min\{1,t\}$ and $\frac{g(1)}{2} \leq Z_i(x) \leq M$, where $M = \sup_{t>0} g(t)$.*

Proof. If $t \geq 1$ then $g(t) \geq g(1) = g(1)\min\{1,t\}$ by the monotonicity of g. If $t < 1$ then $g(t) = tg(1/t) \geq tg(1) = g(1)\min\{1,t\}$ by $g(t) = tg(1/t)$ and the monotonicity of g. From $Z_i(x) = \int_{\mathbb{R}} g(e^{w_i U_i(x)})\mu_\sigma(w)dw$ and $g(t) \leq M$ it follows $Z_i(x) \leq M$. If $U_i(x) \leq 0$, then $g(e^{w_i U_i(x)}) \geq g(1)$ for all $w \leq 0$ and thus $Z_i(x) \geq \int_{-\infty}^0 g(1)\mu_\sigma(w)dw = \frac{g(1)}{2}$. The case $U_i(x) \geq 0$ is analogous. □

Lemma 3.6. *If g is bounded and non-decreasing, then $Z_i(x) \to \frac{M}{2}$ as $U_i(x) \to -\infty$ or $U_i(x) \to +\infty$ and for all $w_i \in \mathbb{R}$ it holds*

$$\begin{aligned} q_i(w_i;x) &\to 2\mu_\sigma(w_i)\,\mathbb{I}_{(-\infty,0]}(w_i) \qquad \text{as } U_i(x) \to -\infty \quad \text{and} \\ q_i(w_i;x) &\to 2\mu_\sigma(w_i)\,\mathbb{I}_{[0,+\infty)}(w_i) \qquad \text{as } U_i(x) \to +\infty\,. \end{aligned}$$

Proof. Consider the case $U_i(x) \to -\infty$. From $g(t) = t\,g(1/t) \le tM$ it follows $g(t) \to 0$ as $t \to 0$. Also, from the boundedness and monotonicity of g it holds $g(t) \to M$ as $t \to \infty$. Therefore, for all $w_i \in \mathbb{R}$,

$$g(\exp(w_i U_i(x))) \to M\,\mathbb{I}_{(-\infty,0]}(w_i) \qquad \text{as } U_i(x) \to -\infty\,. \tag{3.16}$$

Thus, from the bounded convergence theorem $Z_i(x) \to \int_{-\infty}^{0} M\ \mu_\sigma(w_i)dw_i = \frac{M}{2}$ as $U_i(x) \to -\infty$ and, consequently, $q_i(w_i;x) \to 2\mu_\sigma(w_i)\,\mathbb{I}_{(-\infty,0]}(w_i)$ as $U_i(x) \to -\infty$. The case $U_i(x) \to +\infty$ is analogous. □

We now provide two lemmas that will be used to prove the inequality in (3.12).

Lemma 3.7. *Suppose Condition 4 holds. Let* $V_s(x) = \exp(s\|x\|_\infty)$ *and* Q *the kernel with density* q *as in* (3.15). *Then*

$$\inf_{s>0} \limsup_{\|x\|\to\infty} \frac{QV_s(x)}{V_s(x)} = 0\,.$$

Proof. Let $x \in \mathbb{R}^d$ and $Y \sim Q(x,\cdot)$. Since $V_s(y) \le \sum_{i=1}^d \exp(s|y_i|)$ we have

$$\mathbb{E}\left[\frac{V_s(Y)}{V_s(x)}\right] \le \sum_{i=1}^{d} \mathbb{E}\left[\frac{e^{s|Y_i|}}{e^{s\|x\|_\infty}}\right].$$

We now bound $\mathbb{E}\left[e^{s(|Y_i|-\|x\|_\infty)}\right]$ differently depending on whether $|x_i| \le \frac{1}{2}\|x\|_\infty$ or $\frac{1}{2}\|x\|_\infty < |x_i| \le \|x\|_\infty$.

If $|x_i| \le \frac{1}{2}\|x\|_\infty$ it follows from the triangle inequality that $|x_i + w| - \|x\|_\infty \le |x_i| + |w| - \|x\|_\infty \le |w| - \|x\|_\infty/2$ for any $w \in \mathbb{R}$. Also, from (3.15) and Lemma 3.5 we have $q_i(w_i;x) \le \frac{2M}{g(1)}\mu_\sigma(w_i)$. It follows

$$\mathbb{E}\left[e^{s(|Y_i|-\|x\|_\infty)}\right]\mathbb{I}\left(|x_i| \le \frac{\|x\|_\infty}{2}\right) \le \frac{2M}{g(1)} e^{-s\|x\|_\infty/2} \int_{\mathbb{R}} e^{s|w|}\mu_\sigma(w)dw\,,$$

and thus

$$\limsup_{\|x\|\to\infty} \mathbb{E}\left[e^{s(|Y_i|-\|x\|_\infty)}\right]\mathbb{I}\left(|x_i| \le \frac{\|x\|_\infty}{2}\right) = 0\,. \tag{3.17}$$

If $\frac{1}{2}\|x\|_\infty < |x_i| \le \|x\|_\infty$ we have

$$\mathbb{E}\left[e^{s(|Y_i|-\|x\|_\infty)}\right]\mathbb{I}\left(|x_i| > \frac{\|x\|_\infty}{2}\right) \le \mathbb{I}\left(|x_i| > \frac{\|x\|_\infty}{2}\right)\int_{\mathbb{R}} e^{s(|x_i+w|-|x_i|)}q_i(w;x)dw\,.$$

If $\|x\| \to \infty$ and $|x_i| > \frac{\|x\|_\infty}{2}$ it follows $|x_i| \to \infty$. Moreover, by Condition 4 and $|x_i| > \frac{\|x\|_\infty}{2}$, we have $U_i(x) \le \frac{f(\|x\|)}{2} \to -\infty$ as $x_i \to +\infty$ and $U_i(x) \ge -\frac{f(\|x\|)}{2} \to +\infty$ as $x_i \to -\infty$. Therefore, by Lemma 3.6

$$\limsup_{\|x\|\to\infty} \mathbb{I}\left(|x_i| > \frac{\|x\|_\infty}{2}\right)\int_{\mathbb{R}} e^{s(|x_i+w|-|x_i|)}q_i(w;x)dw \le 2\int_{-\infty}^{0} e^{sw}\mu_\sigma(w)dw\,.$$

Combining the last two displayed equations we get

$$\limsup_{\|x\|\to\infty} \mathbb{E}\left[e^{s(|Y_i|-\|x\|_\infty)}\right]\mathbb{I}\left(|x_i| > \frac{\|x\|_\infty}{2}\right) \le 2\int_{-\infty}^{0} e^{sw}\mu_\sigma(w)dw\,. \tag{3.18}$$

From (3.17), (3.18) and basic properties of the lim sup we get

$$\limsup_{\|x\|\to\infty} \mathbb{E}\left[e^{s(|Y_i|-\|x\|_\infty)}\right] \le 2\int_{-\infty}^{0} e^{sw}\mu_\sigma(w)dw\,.$$

Thus

$$\limsup_{\|x\|\to\infty} \mathbb{E}\left[\frac{V_s(Y)}{V_s(x)}\right] \le d\left(\int_{-\infty}^{0} e^{sw}2\mu_\sigma(w)dw\right)$$

which goes to 0 as $s\to\infty$. □

LEMMA 3.8. *Assume that* $\inf_{w\in(-\delta,\delta)}\mu_\sigma(w) > 0$ *for some* $\delta > 0$. *Under Condition 4 it holds*

$$\liminf_{\|x\|\to\infty} \int_{\mathbb{R}^d} q(x,y)\alpha(x,y)dy > 0\,. \tag{3.19}$$

PROOF. Let $w = y - x$ and $\mu_\sigma(w) = \prod_{i=1}^d \mu_\sigma(w_i)$. Also, denote by $\alpha(w;x) = \alpha(x,y)$ the MH acceptance rate when moving from x to y. We write $f(w;x) \gtrsim g(w;x)$ if the function $f(w;x)$ is greater or equal than $g(w;x)$ up to positive constants independent of x and w. From Lemma 3.5 we have $\frac{g(1)}{2} \le Z_i(x) \le M$ and thus

$$\begin{aligned} &q(w;x)\alpha(w;x)\\ &= \frac{\mu_\sigma(w)}{\prod_{i=1}^d Z_i(x)}\min\left\{\prod_{i=1}^d g(e^{w_iU_i(x)}), e^{U(x+w)-U(x)}\prod_{i=1}^d \frac{g(e^{-w_iU_i(x+w)})Z_i(x)}{Z_i(x+w)}\right\}\\ &\gtrsim \mu_\sigma(w)\min\left\{\prod_{i=1}^d g(e^{w_iU_i(x)}), e^{U(x+w)-U(x)}\prod_{i=1}^d g(e^{-w_iU_i(x+w)})\right\}\,. \end{aligned}$$

Then, using $g(t) \ge g(1)\min\{1,t\}$ from Lemma 3.5 we obtain

$$\begin{aligned} &q(w;x)\alpha(w;x)\\ &\gtrsim \mu_\sigma(w)\min\left\{\prod_{i=1}^d g(e^{w_iU_i(x)}), g(1)^d e^{U(x+w)-U(x)+\sum_{i=1}^d \min\{-w_iU_i(x+w),0\}}\right\}\\ &\gtrsim \mu_\sigma(w)\min\left\{\prod_{i=1}^d g(e^{w_iU_i(x)}), e^{U(x+w)-U(x)+\sum_{i=1}^d \min\{-w_iU_i(x+w),0\}}\right\}\,. \end{aligned}$$

Assume $\|x\|$ large and $w\in A(x)$, where $A(x) = \{w\in\mathbb{R}^d : \|x+w\| \le \|x\|-\epsilon,\ \|w\| \le 2\epsilon$ and $x_iw_i \le 0$ for all $i\}$ for some fixed $\epsilon > 0$. From $x_iw_i \le 0$ it follows $w_iU_i(x) \ge 0$ and thus, from the monotonicity of g, $g(e^{w_iU_i(x)}) \ge g(1)$. Combining the latter with the last displayed equation we have

$$q(w;x)\alpha(w;x) \gtrsim \mu_\sigma(w)\min\left\{g(1)^d, e^{U(x+w)-U(x)+\sum_{i=1}^d \min\{-w_iU_i(x+w),0\}}\right\}\,. \tag{3.20}$$

We now lower bound $U(x+w)-U(x)+\sum_{i=1}^d \min\{-w_i U_i(x+w),0\}$. For $\|x\|>R$, from Condition 4

$$\begin{aligned} &U(x+w)-U(x)+\sum_{i=1}^d \min\{-w_i U_i(x+w),0\} \\ &= f(\|x+w\|)-f(\|x\|)+\frac{f'(\|x+w\|)}{\|x+w\|}\sum_{i=1}^d \min\{-w_i(x_i+w_i),0\}\,. \end{aligned}$$

Using the non-increasingness of f' and $w\in A(x)$ we have $f(\|x+w\|)-f(\|x\|)\geq -f'(\|x+w\|)(\|x\|-\|x+w\|)\geq -f'(\|x+w\|)\epsilon$. Thus

$$\begin{aligned} &U(x+w)-U(x)+\sum_{i=1}^d \min\{-w_i U_i(x+w),0\} \\ &\geq -f'(\|x+w\|)\left(\epsilon+\frac{\sum_{i=1}^d \min\{-w_i(x_i+w_i),0\}}{\|x+w\|}\right)\,. \end{aligned}$$

Since $w\in A(x)$ it follows $x_i w_i\leq 0$ and $\min\{-w_i(x_i+w_i),0\}\geq -w_i^2\geq -(2\epsilon)^2$. Thus

$$\inf_{w\in A(x)}\frac{\sum_{i=1}^d \min\{-w_i(x_i+w_i),0\}}{\|x+w\|}\geq -\frac{(2\epsilon)^2}{\|x\|-2\epsilon}\,,$$

which goes to 0 as $\|x\|\to\infty$. It follows that

$$\begin{aligned} &\liminf_{\|x\|\to\infty}\inf_{w\in A(x)} U(x+w)-U(x)+\sum_{i=1}^d \min\{-w_i U_i(x+w),0\}\geq \\ &\liminf_{\|x\|\to\infty} -f'(\|x\|-2\epsilon)\epsilon=\infty\,. \end{aligned}$$

Combining the last displayed equation with (3.20) we have

$$\liminf_{\|x\|\to\infty}\inf_{w\in A(x)} q(w;x)\alpha(w;x)\gtrsim \liminf_{\|x\|\to\infty}\inf_{w\in A(x)}\mu_\sigma(w)>0\,,$$

where the last inequality holds for sufficiently small ϵ because of the assumption $\inf_{w\in(-\delta,\delta)}\mu_\sigma(w)>0$. Therefore

$$\begin{aligned} \liminf_{\|x\|\to\infty}\int_{\mathbb{R}^d} q(x,y)\alpha(x,y)dy &\geq \liminf_{\|x\|\to\infty}\int_{A(x)} q(w;x)\alpha(w;x)dw \\ &\gtrsim \liminf_{\|x\|\to\infty}\int_{A(x)} 1\,dw\,. \end{aligned}$$

The proof is completed noting that $\liminf_{\|x\|\to\infty}\int_{A(x)} 1\,dw>0$ by the construction of $A(x)$. □

PROOF (PROOF OF THEOREM 6). Lemmas 3.7 and 3.8 imply that there exist an $s>0$ such that V_s satisfy (3.12). The thesis then follows from Lemma 3.4, noting that

compact sets are small for P (which can be deduced from the fact that $\inf \pi(x) > 0$ on compact sets) and that that $\sup_x QV_s(x)/V_s(x) < \infty$ because

$$\frac{QV_s(x)}{V_s(x)} \leq \sum_{i=1}^{d} \int_{\mathbb{R}} e^{s|w_i|} q_i(w_i;x)dw_i \leq 2d \int_{\mathbb{R}} e^{s|w_i|} \mu_\sigma(w_i)dw_i < \infty$$

where we used $e^{\|y\|_\infty - \|x\|_\infty} \leq e^{\|y-x\|_\infty} \leq \sum_i e^{|y_i - x_i|}$, $q_i(w_i;x) \leq 2\mu_\sigma(w_i)$ and $\int_{\mathbb{R}} \exp(s|w|)\mu_\sigma(w)dw \leq 2\int_{\mathbb{R}} \exp(sw)\mu_\sigma(w)dw < \infty$ for every $s > 0$. □

3.4. Proof of Proposition 6

Proposition 6 follows directly from Lemmas 3.9 and 3.10 below.

LEMMA 3.9. *Under the assumptions of Proposition 6 we have*

$$\log\left(\frac{f(x_i+\sigma u_i)}{f(x_i)} \frac{g\left(e^{-\phi'(x_i+\sigma u_i)\sigma u_i}\right)}{g\left(e^{\phi'(x_i)\sigma u_i}\right)}\right) = \mathcal{O}\left(\sigma^3\right) \qquad \text{as } \sigma \to 0\,, \tag{3.21}$$

for all $x_i, w_i \in \mathbb{R}$.

PROOF. Define the function b as $b(s) = \log(g(\exp(s)))$ for all $s \in \mathbb{R}$. For any x_i, u_i in $\mathbb{R}$, we have

$$\begin{aligned}
&\log\left(\frac{f(x_i+\sigma u_i)}{f(x_i)} \frac{g\left(e^{-\phi'(x_i+\sigma u_i)\sigma u_i}\right)}{g\left(e^{\phi'(x_i)\sigma u_i}\right)}\right) \\
&= \phi(x_i+\sigma u_i) - \phi(x_i) + b(-\phi'(x_i+\sigma u_i)\sigma u_i) - b(\phi'(x_i)\sigma u_i) \\
&= c_1(x_i)\phi'(x_i)u_i\sigma + c_2(x_i)\frac{u_i^2\sigma^2}{2} + c_3(x_i)\frac{u_i^3\sigma^3}{6} + \mathcal{O}\left(\sigma^4\right) \quad \text{as } \sigma \to 0\,,
\end{aligned} \tag{3.22}$$

where $c_1(x_i)$ and $c_2(x_i)$ are the coefficients of the second order Taylor expansion about $\sigma = 0$, and are given by $c_1(x_i) = (1-2b'(0))\,\phi'(x_i)$ and $c_2(x_i) = (1-2b'(0))\,\phi''(x_i)$. To conclude, we now show that the assumptions on g imply $b'(0) = 1/2$ and $c_1(x_i) = c_2(x_i) = 0$. By definition of b it holds that $b'(0) = g'(1)/g(1)$. From $g(t) = t\,g(1/t)$ it follows $g(1+\epsilon) = (1+\epsilon)\,g((1+\epsilon)^{-1})$ and thus $\frac{g(1+\epsilon)-g((1+\epsilon)^{-1})}{2\epsilon} = \frac{g((1+\epsilon)^{-1})}{2}$. Taking the limit $\epsilon \downarrow 0$ and using $(1+\epsilon)^{-1} = 1-\epsilon+\mathcal{O}(\epsilon^2)$ it follows that $g'(1) = \frac{g(1)}{2}$ and thus $b'(0) = 1/2$ and $c_1(x_i) = c_2(x_i) = 0$. Combining the latter with (3.22) we obtain (3.21). □

REMARK 3.1. *For general* ϕ, x_i *and* u_i, *we have* $\log(\alpha_i(x_i, x_i+\sigma u_i)) = \Theta(\sigma^3)$ *because the third coefficient in the Taylor expansion in* (3.22), *which is given by*

$$c_3(x_i) = 6b''(0)\phi'(x_i)\phi''(x_i) - 2b'''(0)\phi'(x_i)^3 + (1-3b'(0))\phi'''(x_i)\,,$$

is non-zero in general.

LEMMA 3.10. *Under the assumptions of Proposition 6 we have*

$$\log\left(\frac{Z_i(x_i)}{Z_i(x_i+\sigma u_i)}\right) = \mathcal{O}\left(\sigma^3\right) \qquad \text{as } \sigma \to 0\,,$$

for all $x_i, w_i \in \mathbb{R}$.

PROOF. Without loss of generality, assume $g(1) = 1$ throughout the proof. First consider $\log(Z_i(x_i))$, which can be written as

$$Z_i(x_i) = \int_{\mathbb{R}} g\left(e^{\phi'(x_i)(y_i-x_i)}\right)\sigma^{-1}\mu\left(\frac{y_i-x_i}{\sigma}\right)dy_i = \int_{\mathbb{R}} g\left(e^{\phi'(x_i)\sigma s}\right)\mu(s)ds\,. \tag{3.23}$$

For every non-negative integer j, denote by κ_j the j-th moment of the distribution $\mu(\cdot)$. Note that, since μ is a symmetric pdf, $\kappa_0 = 1$, $\kappa_j = 0$ if j is odd and $\kappa_j > 0$ if j is even. For $j \in \{1,2,3\}$, we have

$$\begin{aligned}\frac{\partial^j}{\partial\sigma^j}Z_i(x_i)\Big|_{\sigma=0} &= \int_{\mathbb{R}}\frac{\partial^j}{\partial\sigma^j}g\left(e^{\phi'(x_i)\sigma s}\right)\Big|_{\sigma=0}\mu(s)ds = \\ &\int_{\mathbb{R}}\frac{\partial^j}{\partial\sigma^j}g\left(e^{\phi'(x_i)\sigma}\right)\Big|_{\sigma=0}s^j\mu(s)ds = \frac{\partial^j}{\partial\sigma^j}g\left(e^{\phi'(x_i)\sigma}\right)\Big|_{\sigma=0}\kappa_j\,,\end{aligned} \tag{3.24}$$

where the exchange of integration and derivation is justified by the assumptions on g and μ. Using the Taylor expansion of the function $\sigma \mapsto \log(h(\sigma))$ for general h about $\sigma = 0$, and the fact that $Z_i(x_i)\Big|_{\sigma=0} = 1$ and $\frac{\partial^j}{\partial\sigma^j}Z_i(x_i)\Big|_{\sigma=0} = 0$ if j is odd, we have

$$\begin{aligned}\log(Z_i(x_i)) &= \kappa_2\frac{\partial^2}{\partial\sigma^2}g\left(e^{\phi'(x_i)\sigma}\right)\Big|_{\sigma=0}\frac{\sigma^2}{2} + \mathcal{O}(\sigma^4) \\ &= \kappa_2(g'(1)+g''(1))\phi'(x_i)^2\frac{\sigma^2}{2} + \mathcal{O}(\sigma^4) \quad \text{as } \sigma\to 0\,.\end{aligned} \tag{3.25}$$

Set $y_i = x_i + \sigma u_i$, then from (3.23) and (3.24)

$$\frac{\partial^j}{\partial\sigma^j}Z_i(x_i+\sigma u_i)\Big|_{\sigma=0} = \int_{\mathbb{R}}\frac{\partial^j}{\partial\sigma^j}g\left(e^{\phi'(x_i+\sigma u_i)\sigma s}\right)\Big|_{\sigma=0}\mu(s)ds$$

Reordering the Taylor expansion of $g\left(e^{\phi'(x_i+\sigma u_i)\sigma s}\right)$ about $\sigma = 0$ as a polynomial of s and keeping only even powers in s we get

$$Z_i(x_i+\sigma u_i) = 1 + \kappa_2(g'(1)+g''(1))\phi'(x_i)^2\frac{\sigma^2}{2} + \mathcal{O}(\sigma^3)\,.$$

Using the expansion of $\log(h(\sigma))$ for general h about $\sigma = 0$, and the fact that $Z_i(x_i + \sigma u_i)\Big|_{\sigma=0} = 1$ and $\frac{\partial}{\partial\sigma}Z_i(x_i+\sigma u_i)\Big|_{\sigma=0} = 0$, we have

$$\log(Z_i(x_i+\sigma u_i)) = \kappa_2(g'(1)+g''(1))\phi'(x_i)^2\frac{\sigma^2}{2} + \mathcal{O}(\sigma^3)\,.$$

Combining the latter equation with (3.25) we have

$$\log\left(\frac{Z_i(x_i)}{Z_i(x_i+\sigma u_i)}\right) = \log\left(Z_i(x_i)\right) - \log\left(Z_i(x_i+\sigma u_i)\right) = \mathcal{O}(\sigma^3)$$

□

REMARK 3.2. *For the Barker proposal, the normalization term $Z_i(x_i)$ is constant over x_i and thus Lemma 3.10 is trivially satisfied.*

4. Condition 3 for the exponential family class

PROPOSITION 4.1. *Condition 3 holds in the case in which there are $\alpha, \beta > 0$ such that*

$$\pi(x) \propto \exp\{-\alpha\|x\|^\beta\}$$

PROOF. Condition (ii) is immediate. For (i), first note that here

$$\left|\frac{\partial \log \pi(x)}{\partial x_1}\right| \|x\|^\gamma = -\alpha\beta x_1 \|x\|^{\gamma+\beta-2}. \tag{4.1}$$

Note that $\|x\| = \sqrt{(\sum_i x_i^2)}$ is a monotonically increasing function in each $|x_i|$, so the infimum over $(x_2, ..., x_d)$ of (4.1) is realised at $x_2 = ... = x_d = 0$. Choosing $\gamma = 2$ condition (i) is satisfied because

$$\liminf_{|x_1|\to\infty} \alpha\beta|x_1|^{1+\beta} = \infty.$$

□

5. First-order exact Metropolis-Hastings proposals

Intuitively, we would like any method that uses gradient information to be exact at the first order. In a Metropolis-Hastings context, this means a proposal that incorporates gradient information should be reversible with respect to measures that possess a log-linear density function, i.e. $\pi(x) = \exp(ax + b)$ for some $a, b \in \mathbb{R}$. In such cases the gradient at any location encompasses full information and this would therefore seem to be a sensible minimal goal for well-designed gradient-based methods. The Langevin and Hamiltonian schemes both satisfy this stipulation. As the following proposition shows, for any instance of the class defined in (15), the condition $g(t) = tg(1/t)$ is both sufficient and necessary for the proposal distribution to satisfy such a requirement.

PROPOSITION 5.1. *Let μ_σ be a symmetric probability density function on $\mathbb{R}$ and $\pi(x) = \exp(ax + b)$ for some $a, b \in \mathbb{R}$, with $a \neq 0$. Then a transition kernel of the form in (15) is π-reversible if and only if $g(t) = tg(1/t)$ for every $t > 0$.*

PROOF. Since $\nabla \log \pi(x) = a$ for every $x \in \mathbb{R}$, it follows that the normalizing constant, Z, of $q(x, y)$ in (15) is independent of x. First we show that $g(t) = tg(1/t)$ implies reversibility. From the symmetry of μ_σ and $g(t) = tg(1/t)$ it follows

$$\begin{aligned}\pi(x)q(x,y) &= \exp(ax+b)Z^{-1}g\left(\exp\left(a(y-x)\right)\right)\mu_\sigma(x,y)\\ &= \exp(ax+b)Z^{-1}\exp\left(a(y-x)\right)g\left(\exp\left(-a(y-x)\right)\right)\mu_\sigma(y,x)\\ &= \pi(y)q(y,x)\,,\end{aligned}$$

which implies that q is π-reversible. Conversely, if q is π-reversible, then

$$1 = \frac{\pi(x)q(x,y)}{\pi(y)q(y,x)} = \frac{\exp(a(x-y))g\left(1/\exp\left(a(x-y)\right)\right)}{g\left(\exp\left(a(x-y)\right)\right)} = \frac{tg(1/t)}{g(t)}\,,$$

for $t = \exp(a(x-y))$. For $a \neq 0$, $\exp(a(x-y))$ takes any positive value as $x, y \in \mathbb{R}^d$ and thus we have $g(t) = tg(1/t)$ for every $t > 0$. □

REMARK 5.1. *Note that $\pi(x) = \exp(ax+b)$ is an improper density function because $\int_{\mathbb{R}} \exp(ax+b)dx = \infty$ for any choice of a and b. This, however, does not pose any issue in defining π-reversible kernels as usual.*

6. Locally balanced proposals and skew-symmetric distributions

In this section we show that the only balancing function g leading to a skew-symmetric distribution is $g(t) = t/(1+t)$. Following (Azzalini, 2013), a skew-symmetric distribution on $\mathbb{R}$ is distribution for which the probability density can be written

$$f(z) = 2f_0(z)G(z),$$

for any $z \in \mathbb{R}$, where $f_0(z) = f_0(-z)$, $G(z) \geq 0$ and

$$G(z) + G(-z) = 1. \tag{6.1}$$

In the first-order locally-balanced framework, if the current point is x then the proposal has density

$$f_x(z) = Z(x)^{-1}\mu_\sigma(z)g(e^{\nabla \log \pi(x) z}),$$

where, setting $t = e^{\nabla \log \pi(x) z}$ the balancing function g satisfies

$$g(t) = tg(1/t). \tag{6.2}$$

Equating (6.1) and (6.2) gives $G(z) = g(e^{\nabla \log \pi(x) z}) = g(t)$, implying that in this case

$$G(-z) = g(1/t).$$

Therefore, dividing (6.1) by $G(1/z)$, using the above and combining with (6.2) gives

$$t + 1 = \frac{1}{g(1/t)},$$

and combining with (6.2) gives

$$g(t) = \frac{t}{1+t}.$$

as required.

7. Pre-conditioning the Barker proposal

The diagonal non-isotropic version of the Barker scheme (corresponding to using a diagonal preconditioning matrix) is a simple variation of Algorithm 2 from the paper and is described in Algorithm 7.1. The acceptance probability related to Algorithm 7.1 is exactly the same $\alpha^B(x, y)$ defined in (18) of the paper.

Algorithm 7.1 Diagonal Barker proposal on $\mathbb{R}^d$

Require: current point $x \in \mathbb{R}^d$ and local scales $(\sigma_1, \ldots, \sigma_d) \in (0, \infty)^d$
Independently for each $i \in \{1, \ldots, d\}$ do:

(a) Draw $z_i \sim \mu_{\sigma_i}$
(b) Calculate $p_i(x, z_i) = 1/(1 + e^{-z_i \partial_i \log \pi(x)})$
(c) Set $b_i(x, z_i) = 1$ with probability $p_i(x, z_i)$, and $b_i(x, z_i) = -1$ otherwise
(d) Set $y_i = x_i + b_i(x, z_i) \times z_i$

Output: the resulting proposal y.

The general pre-conditioned version of the Barker algorithm is obtained by defining an appropriate linear transformation to the target variables x and then applying the standard Barker algorithm (Algorithm 2 from the paper) in the transformed space. More precisely, given a target π and a covariance matrix Σ with Cholesky factor C, define the transformed variables $\tilde{x} = (C^T)^{-1} x$ with distribution $\tilde{\pi}(\tilde{x}) \propto \pi(C^T \tilde{x})$ and log-gradient $\nabla \log \tilde{\pi}(\tilde{x}) = \nabla \log \pi(C^T \tilde{x}) C^T$. One then applies the standard (isotropic) Barker scheme described in Algorithm 2 of the paper to the pre-conditioned target $\tilde{\pi}$. As typically done with pre-conditioned MALA, the resulting preconditioned Barker scheme can be implemented without explicitly defining the auxiliary variables $\tilde{x}$ and transformed target $\tilde{\pi}$, but rather keeping the original target π and modifying the proposal distribution. The resulting pre-conditioned Barker proposal distribution and corresponding Metropolis-Hastings scheme are described in Algorithms 7.2 and 7.3, respectively.

Algorithm 7.2 Preconditioned Barker proposal on $\mathbb{R}^d$

Require: current point $x \in \mathbb{R}^d$ and preconditioning matrix $C = chol(\Sigma)$.

(a) Draw $z_i \sim \mu$ independently for each $i \in \{1, \ldots, d\}$
(b) Calculate $p_i(x, z) = 1/(1 + e^{-z_i c_i(x)})$ where $c_i(x) = (\nabla \log \pi(x) \cdot C^T)_i$
(c) For each i, set $\tilde{z}_i = z_i$ with probability $p_i(x, z)$, and $\tilde{z}_i = -z_i$ otherwise
(d) Set $y = x + C^T \tilde{z}$ where $\tilde{z} = (\tilde{z}_1, \ldots, \tilde{z}_d)$

Output: the resulting proposal y.

---

8. Additional simulation studies

In this section we provide various additional details on the simulation studies presented in the paper.

Algorithm 7.3 Metropolis–Hastings with preconditioned Barker proposal

Require: starting point for the chain $x^{(0)} \in \mathbb{R}^d$, and preconditioning matrix $C = chol(\Sigma)$.

Set $t = 0$ and do the following:

(a) Given $x^{(t)} = x$, draw y using Algorithm 7.2 and compute

$$\alpha^B(x, y) = \min\left(1, \frac{\pi(y)}{\pi(x)} \times \prod_{i=1}^{d} \frac{1 + e^{-z_i c_i(x)}}{1 + e^{z_i c_i(y)}}\right).$$

where $z_i = ((C^T)^{-1}(y - x))_i$ and $c_i(x) = (\nabla \log \pi(x) \cdot C^T)_i$

(b) Set $x^{(t+1)} = y$ with probability $\alpha^B(x, y)$, and $x^{(t+1)} = x$ otherwise

(c) If $t + 1 < N$, set $t \leftarrow t + 1$ and return to step 1, otherwise stop.

Output: the Markov chain $\{x^{(0)}, \ldots, x^{(N)}\}$.

8.1. Additional example for Section 5.1 of the paper

In Figure 8.1 we display a phenomenon analogous to Figure 2 of the paper on a 20-dimensional example in which each component of $\pi(\cdot)$ is an independent $N(0, \eta_i^2)$ random variable, with $\eta_1 = 0.01$ and $\eta_i = 1$ for $i = 2, ..., 20$. Here the performance of MALA starts deteriorating drastically as soon as the step-size exceeds the scale of the first component as we would expect from the theory developed in Section 2 of the paper. On the other hand both the random walk and Barker schemes can function adequately with larger than optimal step-sizes, and as a result achieve a much higher expected squared jump distance on all the other coordinates.

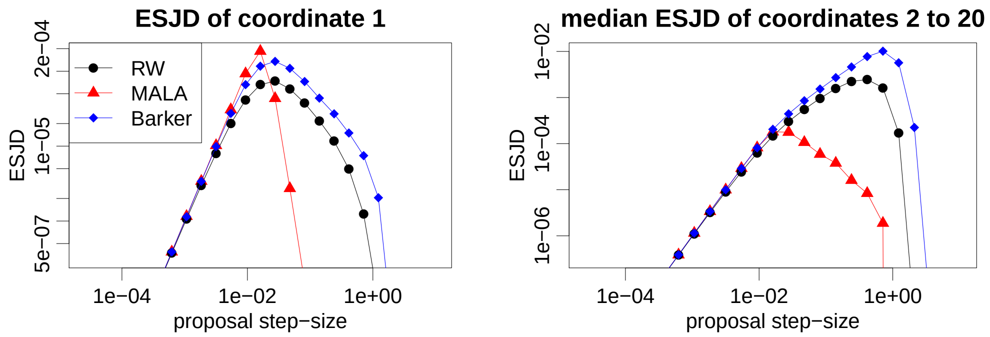

Fig. 8.1. Expected squared jump distance (ESJD) against proposal step-size for RW, MALA and Barker on a 20-dimensional target in which one component has a smaller scale than all others.

8.2. Traceplots for Scenarios 2-4 from Section 6.2 of the paper

Figure 4 of the paper displays the evolution of tuning parameters and MCMC trajectories when targeting the distribution described in Scenario 1 of that section. Here we provide

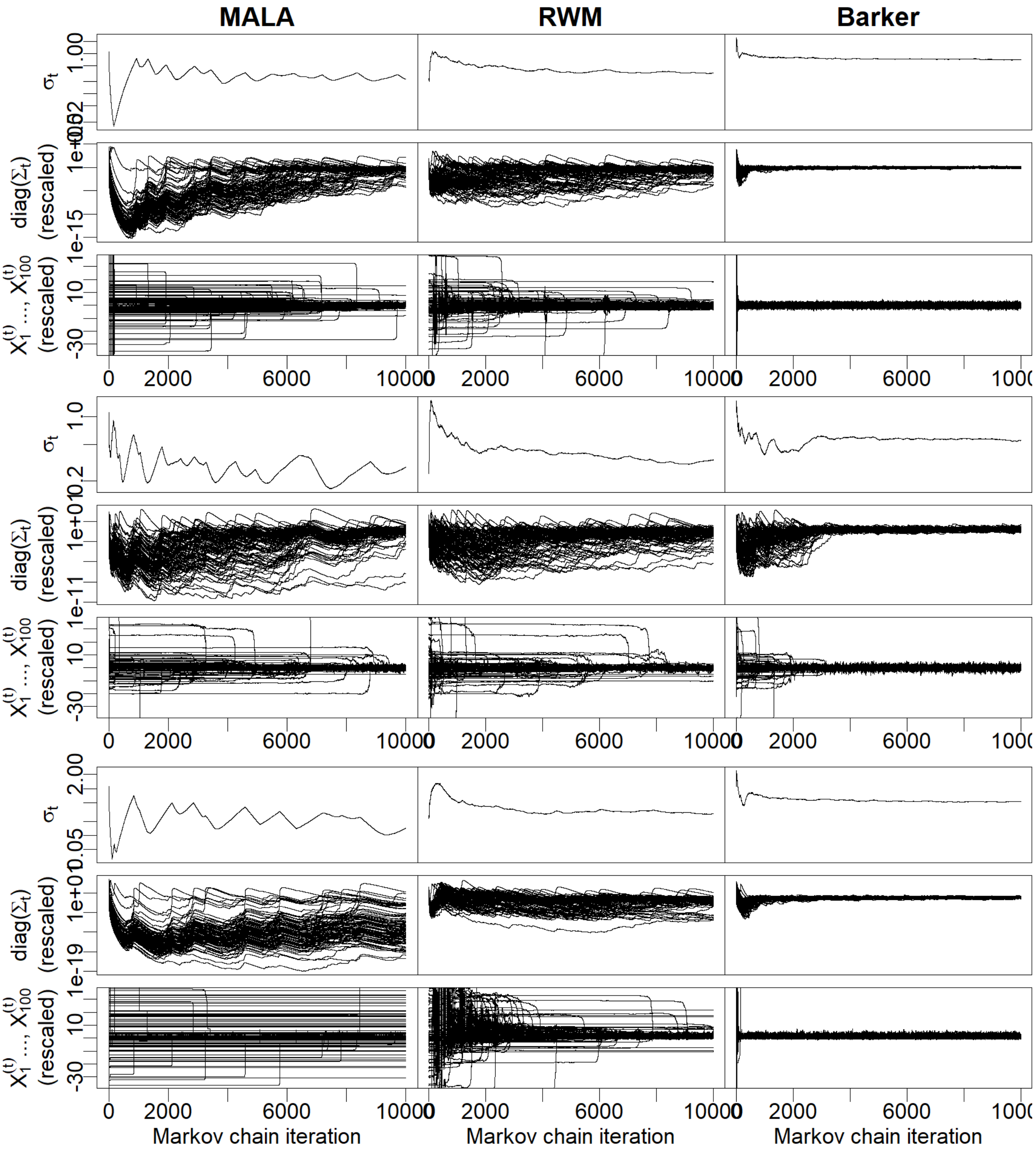

Fig. 8.2. Same as Figure 4 of the paper for the target distributions of Scenario 2 (rows 1 to 3), Scenario 3 (rows 4 to 6) and Scenario 4 (rows 7 to 9). For each scenario, the first row displays the traceplot of the global scale σ_t; the second row the ones of the *normalized* local scales $\Sigma_{t,ii}/\Sigma_{ii}$ for $i = 1, \dots, 100$; and the third row the ones of the *normalized* coordinates $X_i^{(t)}/\Sigma_{ii}^{1/2}$ for $i = 1, \dots, 100$. See Section 6.2 of the paper for more details.

analogous illustrations for Scenarios 2, 3 and 4. Figure 8.2 displays, for each scenario and each algorithm, the traceplot of the global scale $(\sigma_t)_{t\geq 1}$, the ones of the normalized local scales $(\Sigma_{t,ii}/\Sigma_{ii})_{t\geq 1}$ for $i \in \{1,\dots,100\}$ and the ones of the normalized Markov chains coordinates $(X_i^{(t)}/\Sigma_{ii}^{1/2})_{t\geq 1}$ for $i \in \{1,\dots,100\}$. Here Σ is the covariance of the target distribution and normalization is used to facilitate readability, so that all normalized local scales converge to 1 as $t \to \infty$ and all normalized coordinates have a $N(0,1)$ limiting distribution as $t \to \infty$. Overall, the traceplots for Scenarios 2-4 display a qualitatively similar behaviour to the ones of Scenario 1 Figure 4 of the paper. See Section 6.2 of the paper for more discussion.

8.3. Comparison to truncated or tamed gradients

Consider Metropolis–Hastings proposals of the form

$$y = x + \frac{\sigma^2}{2}G(x) + \sigma\xi\,,$$

for some $\sigma > 0$, $G : \mathbb{R}^d \to \mathbb{R}^d$ and $\xi \sim N(0, I)$. Setting $G(x) = \nabla \log \pi(x)$ leads to the MALA proposal. A common way to improve the stability of MALA in the literature is to truncate or tame the gradient $\nabla \log \pi(x)$. For example, in the truncated MALA algorithm (MALTA) (Atchade, 2006) we have

$$G(x) = \frac{\delta}{\max\{\delta, \|\nabla \log \pi(x)\|\}}\nabla \log \pi(x)\,,$$

for some $\delta > 0$, while in the component-wise tamed MALA (MALTAc) (Brosse et al., 2018, eq.(4)) the function $G(x) = (G_1(x),\dots,G_d(x))$ is defined component-wise as

$$G_i(x) = \frac{\partial_i(x)}{1 + \sigma^2|\partial_i(x)|}\,.$$

The above taming is defined in such a way that $|G_i(x)|$ converges to σ^{-2} as $|\partial_i(x)| \to \infty$, meaning that in this case the upper bound for tamed gradients is authomatically chosen in a way that depends on σ.

These schemes are effective in achieving geometric ergodicity also for light tails (Atchade, 2006). They are less effective, however, in terms of being robust to tuning and they are very sensitive to the choice of truncation parameter (respectively δ and σ^{-2}). We illustrate this point in Figure 8.3. There we compare MALTA, MALTAc and Barker on targets being 100-dimensional Gaussian distributions with one component much smaller than the others, analogously to the first scenario of Section 6.2 of the paper. For MALTA we set $\delta = 1000$, as is done for example, in Atchade (2006). We also tried setting $\delta = 100$ without observing major differences. Rows 1-3 of Figure 8.3 consider exactly the same target of Figure 4 of the paper, which is a 100-dimensional Gaussian where the first component standard deviation is equal to 0.01 and all others standard deviations are equal to 1. In this case both MALTA and MALTAc improve over MALA, and in particular that MALTAc manages to converge to stationarity in around 4000 iterations, although this is still significantly slower than Barker (which only

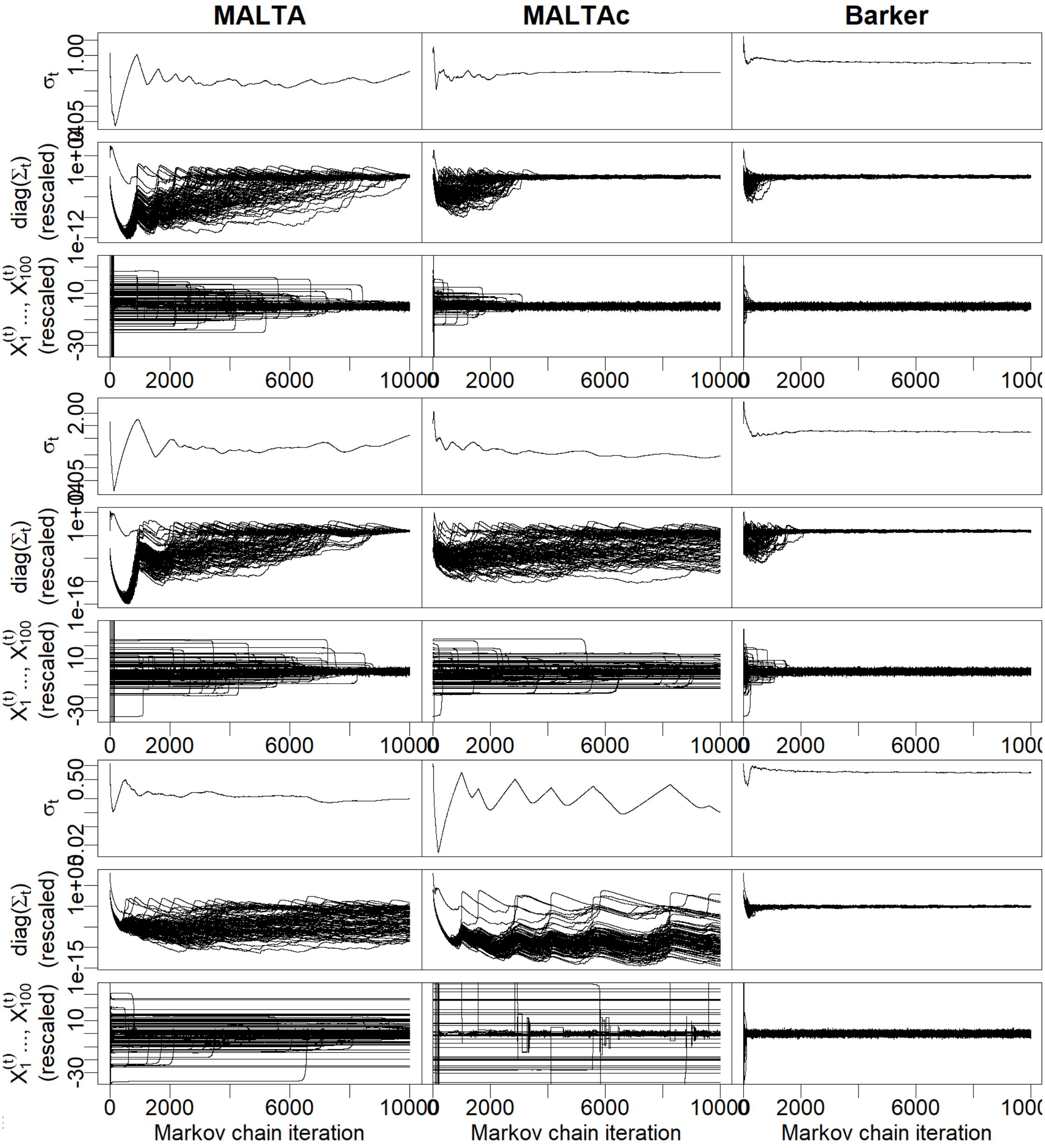

Fig. 8.3. Comparison of MALTA, MALTAc and Barker on target distributions with one small component. Rows 1-3: same target considered in Figure 4 of the paper (100-dimensional Gaussian with first component standard deviation equal to 0.01 and all others standard deviations equal to 1). Rows 4-6 and rows 7-9: re-scaled versions where the scales of all coordinates are either multiplied or divided by 100. See Figure 8.2 for an explanation of each parameter plotted.

requires few hundred iterations). We then consider modifying the target distribution by either multiplying the scales of all coordinates by 100, resulting in the first compo-

nent standard deviation equal to 1 and all others standard deviations equal to 100, or dividing all scales by 100, resulting in the first component standard deviation equal to 10^{-4} and all others standard deviations equal to 10^{-2}. The results are reported in rows 4-6 and rows 7-9 of Figure 8.3, respectively. We observe a dramatic deterioration in the performances of both MALTA and MALTAc, while the performance of the Barker scheme are much less affected. The underlying reason is that MALTA and MALTAc are highly sensitive to the choice of truncation parameter (respectively δ and σ^{-2}), which needs to be tuned appropriately depending on the scales of the target distribution.

These illustrative simulations suggest that ad-hoc strategies to improve the robustness of gradient-based MCMC, such as truncating or taming gradients, are intrinsically more fragile and sensitive to heterogeneity and scales compared to a more principled solution such as the Barker algorithm, in which robustness arises naturally from the proposal mechanism. In addition to this, truncating and taming can be thought of as introducing a 'bias' into the proposal mechanism, in the sense that the resulting proposal is no longer first-order exact. Depending on how the truncation level δ is scaled with the dimensionality d, this can compromise the $d^{-1/3}$ scaling behaviour discussed in the paper.

References

Alzer, H. (1997) On some inequalities for the incomplete gamma function. *Mathematics of Computation of the American Mathematical Society*, **66**, 771–778.

Atchade, Y. F. (2006) An adaptive version for the metropolis adjusted langevin algorithm with a truncated drift. *Methodology and Computing in applied Probability*, **8**, 235–254.

Azzalini, A. (2013) *The skew-normal and related families.* Institute of Mathematical Statistics Monographs. Cambridge University Press.

Brosse, N., Durmus, A., Moulines, É. and Sabanis, S. (2018) The tamed unadjusted langevin algorithm. *Stochastic Processes and their Applications.*

Gautschi, W. (1959) Some elementary inequalities relating to the gamma and incomplete gamma function. *Journal of Mathematics and Physics*, **38**, 77–81.

Jarner, S. F. and Hansen, E. (2000) Geometric ergodicity of Metropolis algorithms. *Stochastic processes and their applications*, **85**, 341–361.

Livingstone, S. and Zanella, G. (2020) The barker proposal: combining robustness and efficiency in gradient-based mcmc. *submitted to the Journal of the Royal Statistical Society: Series B.*

Roberts, G. O. and Tweedie, R. L. (1996) Exponential convergence of langevin distributions and their discrete approximations. *Bernoulli*, **2**, 341–363.

Tierney, L. (1998) A note on Metropolis-Hastings kernels for general state spaces. *The Annals of Applied Probability*, **8**, 1–9.
